# Phytochemical Screening and Isolation of New *Ent*-Clerodane Diterpenoids from *Croton guatemalensis* Lotsy

**DOI:** 10.3390/plants11223159

**Published:** 2022-11-18

**Authors:** Sonia Marlen Escandón-Rivera, Adolfo Andrade-Cetto, Daniel Genaro Rosas-Ramírez, Roberto Arreguín-Espinosa

**Affiliations:** 1 Departamento de Biología Celular, Facultad de Ciencias, Universidad Nacional Autónoma de México, Av. Universidad 3000, Circuito Exterior S/N, Coyoacán, Ciudad Universitaria, Mexico City 04510, Mexico; 2 Departamento de Biomacromoléculas, Instituto de Química, Universidad Nacional Autónoma de México, Av. Universidad 3000, Circuito Exterior S/N, Coyoacán, Ciudad Universitaria, Mexico City 04510, Mexico

**Keywords:** *Croton guatemalensis*, *ent*-clerodane diterpenoid, circular dichroism, HPLC quantification, *α*-glucosidase inhibitor, docking

## Abstract

Phytochemical screening of an ethanol–water extract (EWE) from the bark of *Croton guatemalensis* led to the isolation and identification of eight compounds, among them: five *ent*-clerodane diterpenoids [junceic acid (**1**), 6(*s*)-acetoxy-15,16-diepoxy-*ent*-cleroda-3,13(16),14-trien-20-oic acid (crotoguatenoic acid A) (**2**), 6(*s*)-hydroxyoxy-15,16-diepoxy-*ent*-cleroda-3,13(16),14-trien-20-oic acid (crotoguatenoic acid B) (**3**), formosin F (**4**), bartsiifolic acid (**5**)], and three flavonoids [rutin (**6**), epicatechin (**7**), and quercetin (**8**)]. Of these, **2** and **3** are reported here for the first time. Structures were established through conventional spectroscopy methods and their absolute configurations were determined by optical rotation and comparison of experimental electronic circular dichroism (ECD) and theoretical calculated ECD spectra. A suitable high performance liquid chromatography (HPLC) method for quantifying rutin (**6**) was developed and validated according to standard protocols. Affinity-directed fractionation was used to identify possible in vitro active compounds on *α*-glucosidases from *Saccharomyces cerevisiae*. HPLC-ESI-MS was used to identify the inhibitors as free ligands after being released from the enzymatic complex by denaturing acidic conditions. The affinity studies led to the identification of *ent*-clerodane diterpenoids as active compounds. In silico analysis allowed us to determine the best conformational rearrangement for the *α*-glucosidase inhibitors.

## 1. Introduction

About 1300 species of the Croton genus (Euphorbiaceae) were reported, which are distributed in tropical climate regions around the world [1]. Extracts of different parts of the plant (aerial parts, roots, leaves, bark, etc.) are used for the treatment of various ailments, such as stomachache, abscesses, inflammation, cancer, diabetes, and malaria in the Americas, Africa, and South Asia [1,2,3]; for example, in an in vivo assay of the ethanolic extract of the aerial parts of *C. zambesicus* was performed to determine antiplasmodial activity against chloroquine-sensitive *Plasmodium berghei* infections in mice [4], crude leaf extracts of *Croton cajucara* exhibited a significant antinociceptive effect in rats. The cortex bark is one of the most used parts of pharmacological interest and was studied as an analgesic, anti-inflammatory, antiulcerogenic, gastroprotective, antiviral, antibacterial, antitumor, and hypoglycemic agent [2,3,5]. Many compounds were isolated and identified, among the most important are terpenes, and within them the diterpenes with different types of skeletons are the predominant group, with more than 800 registered compounds, including clerodane, tigliane, kaurane, crotofolane, labdane, cembrane, abietane, casbane, halimane, pimarane, cleistanthane, grayanane, atisane, phytane, and laevinane diterpenoids; of which clerodanes are the most abundant [1,2]. Some alkaloids and phenolic compounds were also isolated. Recent interest in searching for flavonoids in this genus led to the identification of proanthocyanidins, flavones, glycosylated flavonols, and lignans; among the most common are rutin, quercetin, kaempferol, catechin, and epicatechin [6,7,8,9], which could serve as genus chemical markers. Finally, the essential oils of various Croton species present *α*-pinene, *ß*-pinene, camphor, 1,8-cineole, and germacrenes among their most abundant compounds [10,11,12,13,14].

*Croton guatemalensis* Lotsy (Cg) is a small tree, up to 6 m high, distributed in the tropical and subtropical areas of the Americas, including Mexico, Colombia, Ecuador, and Guatemala; it is also known as “copalchi”. Aqueous and methanolic extracts of leaves and cortex were reported as antiplasmodial and cytotoxic [15]; whereas the aqueous extract of the bark is antinociceptive [16]. Our group proved that the aqueous and hydroalcoholic extracts have hypoglycemic activity [17]; so far, no compounds isolated from the plant are reported. In this research, the EWE, such as the one used in the previous work, from the bark of *C. guatemalensis* was fractionated to isolate the main compounds to provide information about its chemical profile; furthermore, with the aim to understand the ecological role of some of the isolated compounds affinity studies for the identification of new α−glucosidase inhibitors were performed.

## 2. Results and Discussion

### 2.1. Isolation and Identification of Previously Undescribed Compounds

**Figure 1:**
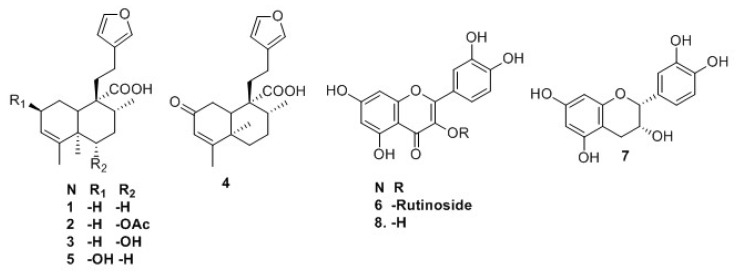
Structure of isolated compounds **1** to **8**.

The ethanol–water extract (EWE) from *C. guatemalensis* was subjected to fractionation procedures to obtain five *ent*-clerodane diterpenes [junceic acid (**1**), 6(*s*)-acetoxy-15,16-diepoxy-*ent*-cleroda-3,13(16),14-trien-20-oic acid (crotoguatenoic acid A) (**2**), 6(*s*)-hydroxyoxy-15,16-diepoxy-*ent*-cleroda-3,13(16),14-trien-20-oic acid (crotoguatenoic acid B) (**3**), formosin F (**4**), bartsiifolic acid (**5**)], and three flavonoids [rutin (**6**), epicatechin (**7**), and quercetin (**8**)] (Figure 1). Of these, two diterpenes were not previously described (**2** and **3**), whereas **1**, **4**, **5**, and **7** were identified based on comparisons of their ^1^H and ^13^C-NMR spectral data, including data obtained in 2D experiments (COSY, HSQC, HMBC, NOESY, and TOCSY) and their mass spectral data, with those of previously described compounds [18,19,20,21]. Flavonoids **6** and **8** were analyzed by HPLC, and their retention time and UV spectra were compared with standards (>94% HPLC; Sigma-Aldrich) of rutin (**6**) and quercetin (**8**), respectively.

**Figure 2:**
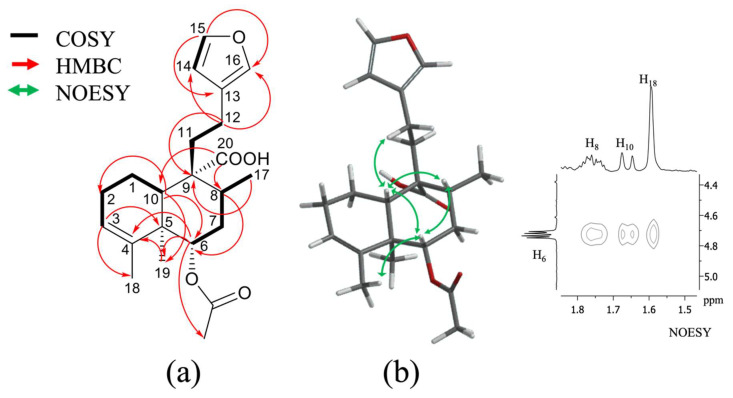
(**a**) ^1^H-^1^H COSY and key HMBC (H—C) correlations of compound **2**; (**b**) Key NOESY correlations of compound **2**.

Compound **2** was obtained as white powder, with a melting point of 96 to 100 °C and molecular formula C_22_H_30_O_5_ derived by NMR spectroscopy data and the ESI-MS ion at 397.4 [M + Na]^+^ and HRESIMS ion at 373.1813 [M–H]^−^ (calcd. for C_22_H_29_O_5_, 373.2020), suggesting eight degrees of unsaturation (Figures S1–S10). The IR spectrum (Figure S11) exhibited a broad absorption band of hydroxyl (3380 cm^−1^), conjugated carbonyl (1714 cm^−1^ of a carboxylic acid and 1693 cm^−1^ of carbonyl ester), and double bond (1408, 1358, and 1372 cm^−1^) functional groups. The ^1^H, ^13^C, and DEPT spectra (Table 1; Supplementary Data, Figures S1–S3) showed the presence of a 22-carbon entity including four methyls, five methylenes, three methines, six vinylic, and four quaternary carbons; ^1^H and ^13^C spectra (Table 1, Figures S1 and S2), HSQC (Figure S4), and COSY (Figure S5) correlations showed typical signs of a clerodane skeleton, diterpene, with three methyl groups, as part of the base skeleton at *δ*_C/H_ 13.9/1.09 (s; C/H-19), 16.1/1.14 (d, *J* = 6.69 Hz; C/H-17), and 21.2/1.59 (br s; C/H-18); and a methyl acetate as a radical at *δ*_C/H_ 22.0/2.04 (s) in the B ring of decalin; two pairs of carbons are part of characteristic signals of a furan ring at *δ*_C/H_ 110.9/6.26 (dd, *J* = 1.83, 0.90 Hz; C/H-14), 124.4 (C-13), 138.7/7.23 (dd, *J* = 1.61, 0.88 Hz; C/H-16), and 143.1/7.35 (t, *J* = 1.69 Hz; C/H-15). The chemical shift of the double bond between *δ*_C/H_ 123.6/5.30 (dd; *J* = 2.87, 1.39 Hz; C/H-3) and *δ*_C_ 141.5 (C-4) indicates the absence of any substituent at C-18. The carboxyl group noted at *δ*_C_ 182.3, which presented HMBC correlation (Figure 2a and Figure S6) with H-10 (*δ*_H_ 1.66; d, *J* = 11.50 Hz) and H-8 (*δ*_H_ 1.76; m), demonstrates its unequivocal position at C-20. The relative configuration of **2** was possible by the NOESY spectrum (Figure S7), and these data showed NOESY correlations (Figure 2b) of H-6 with H-10 and H-8, suggesting the beta position; simultaneously, H-10 with H-12 (*δ*_H_ 2.33; m) and H-19 (*δ*_H_ 1.09; s) with H-7*α* (*δ*_H_ 2.20; m), indicating a *trans-cis*-type clerodane skeleton configuration, as occurs in most of these diterpenes [22]. These signals were similar to those of the isolated *ent*-clerodane diterpene junceic acid (**1**) [18]. However, the main difference was the H-6 deshielded at *δ*_H_ 4.73 (dd, *J* = 11.34, 5.0 Hz), which presented HMBC correlation (Figure 2a) with the acetate moiety (*δ*_C_ 22.0) and TOCSY correlation (Figure S8) with *δ*_H_ 2.20 (m, H-7*α*), 1.76 (m, H-7*β*), and 1.14 (d, *J* = 6.69 Hz, H-17).

**Figure 3:**
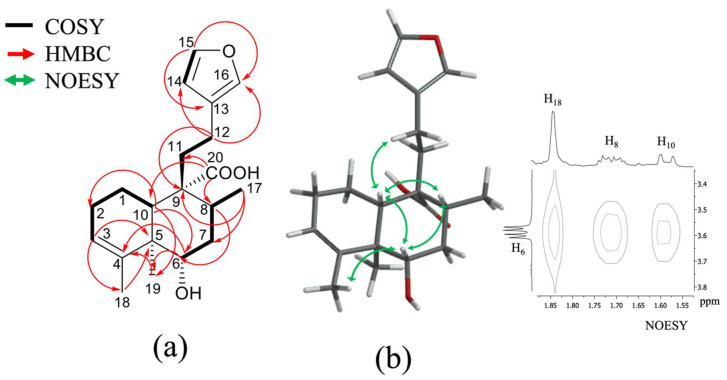
(**a**) ^1^H-^1^H COSY and key HMBC (H—C) correlations of compound **3**; (**b**) key NOESY correlations of compound **3**.

Compound **3** was isolated as white powder with a melting point of 154 to 156 °C and molecular formula C_20_H_28_O_4_ derived by NMR spectroscopy data and the HRESIMS ion at 333.20515 [M + H]^+^ (calcd. for C_20_H_29_O_4_, 333.20658) (Figures S12–S21), suggesting seven degrees of unsaturation. The IR spectrum (Figure S22) exhibited an absorption band of hydroxyl (3399 cm^−1^), conjugated carbonyl (1695 cm^−1^), and double bond (1450 and 1376 cm^−1^) functional groups. The ^1^H- and ^13^C- NMR data of **3** (Table 1, Figures S12 and S13) indicated a diterpenoid of the clerodane class, which was supported by the DEPT, 2D HSQC, COSY, HMBC, NOESY, and TOCSY experiments (Figures S14–S19), **3** showed a close relationship with compound **2** (Table 1). The only difference was the functional group at C-6 with a hydroxyl with chemical shift at *δ*_H_ 3.59 (dd, *J* = 11.35, 4.97 Hz, H-6) instead of an acetate. The position of this hydroxyl was corroborated with the HMBC spectrum (Figure S17), which shows HMBC correlation (Figure 3a) between H-6 with C-10 (*δ*_C_ 47.2), C-4 (*δ*_C_ 143.0), C-19 (*δ*_C_ 12.8), and C-8 (*δ*_C_ 34.7) just as **2**. The relative configurations at C-6 (*δ*_C_ 76.1) and C-10 (*δ*_C_ 47.2) were determined using the NOESY spectrum (Figure S18); the NOESY correlation (Figure 3b) of H-6 with H-10 (*δ*_H_ 1.58; dd, *J* = 11.63, 1.30) and H-8 (*δ*_H_ 1.71; ddd, *J* = 14.06, 6.21, 3.58); simultaneously, H-10 with H-12 (*δ*_H_ 2.33, m), and H-19 (*δ*_H_ 0.98, s) with H-7α (*δ*_H_ 2.20) showed the same *trans-cis*-type clerodane skeleton configuration as **2.**

**Figure 4:**
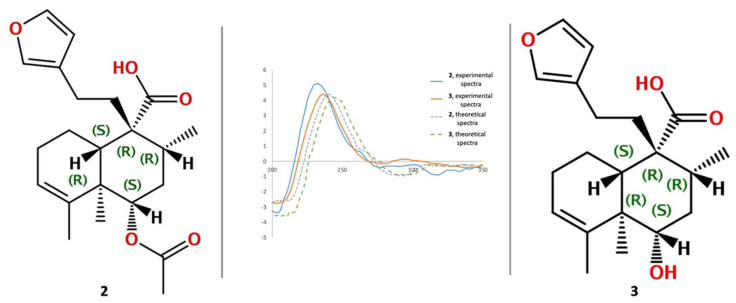
Experimental circular dichroism (CD) spectra for compounds **2** and **3**, in addition to the calculated CD spectra.

To establish the absolute configuration of compounds **2** and **3**, specific rotation and comparison of the experimental and calculated ECD curves for the *ent* isomer (Figure 4) were performed. Specific rotation of **2** ([α]^20^_D_ = −12.0) and **3** ([α]^20^_D_ = −12.3) were of negative value and similar. The experimental ECD spectrum for compound **2** (blue line, Figure 4) and compound **3** (orange line, Figure 4) showed a positive and negative Cotton effect at 236 nm (Δε = +4.40), 204 nm (Δε = −2.73) for **2**, and at 232 nm (Δε = +5.12) and 204 nm (Δε = −3.36) for **3**, which fit with the theoretical ECD spectrum simulated for the *ent* isomer (gray line, ECD theoretical for **2**; green line, ECD theoretical for **3**; Figure 4). Thus, the absolute configuration for compounds **2**–**3** was established as 5R, 6S, 8R, 9R, and 10S stereoisomers.

### 2.2. HPLC Phytochemical Profiling

**Figure 5:**
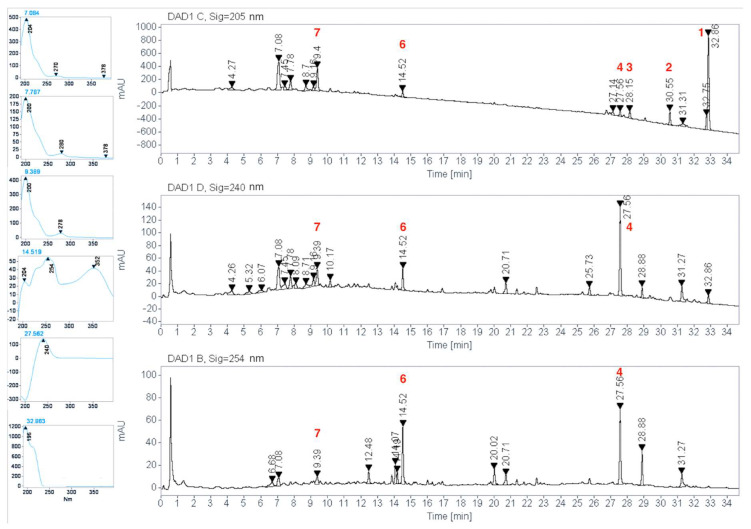
HPLC profile of the EWE of *C. guatemalensis* at 205, 240, and 254 nm and their UV spectra at some peaks at different retention times.

HPLC profile of the EWE of *C. guatemalensis* was monitored at different wavelengths and the peak heights were evaluated. Maximum peak heights for the extract were obtained at 205, 240, and 254 nm (Figure 5) and were selected as optimum wavelengths to analyze the chromatographic profile. The UV spectra of the peaks showed characteristic bands of flavonoids with features of flavans, flavonols, and terpenes, with their maximum absorptions at 200, 266–280 nm (Band II) for flavans [23] in the first 13 min of the profile; then, maximum absorptions at 230–254 (Band II) and 330–370 nm (Band I) for flavonols [23] between 14 and 20 min, and, finally, absorption maxima of 205, 218, and 240 nm for terpenes [24,25] during 20 to 35 min. Isolated compounds were identified in the chromatogram (Figure 5) by coelution of pure compounds or standards with the extract and comparison of their UV spectra. The profile recorded at 205 nm shows the presence of about eight major peaks released in the retention times, 7.08 min (unidentified), 7.78 min (unidentified), 9.39 min (**7**), 14.52 min (**6**), 27.56 min (**4**), 28.15 min (**3**), 30.55 min (**2**), and 32.86 min (**1**); a general qualitative analysis revealed that the most abundant compound was the junceic acid (**1**; 32.86 min; 205 nm) followed by the unidentified peak at 7.08 min, **7** (t_R_ = 9.39; 205 nm), **2** (t_R_ = 30.55 min; 205 nm), **4** (t_R_ = 27.56 min; 240 nm), and **6** (t_R_ = 14.52 min; 254 nm).

Diterpenes are characteristic components of the *Croton* species and clerodane diterpenes skeletons are the most abundant, being part of 27% of the diterpenes found in *Croton* species [1]. Junceic acid (**1**) was first isolated from *Solidago juncea* Ait [18] and previously identified as a major compound in *Croton sarcopetalus* [26] and *Croton arboreus* [27], and it was tested as an anti-inflammatory [28] and phytotoxic [20] agent. Formosin F (**4**) was previously isolated from *Excoecaria formosana* and analyzed as an antibacterial compound that showed moderate antibacterial activity against two strains of *Helicobacter pylori* [19]. Bartsiifolic acid (**5**) was previously isolated from *Blakiella bartsiifolia* [20] and *E. formosana* [19]. It was studied as phytotoxic and antimicrobial. In terms of its phytotoxic activity, it restrained seed germination at low concentration and hindered elongation of the shoots [20]; its antibacterial activity was not proven. Some flavonoids were isolated from various *Croton* species [1]; among these are flavans, flavonol aglycones, flavonol glycosides, flavones, etc. Rutin (**6**) was first described in *Croton menthodorus* [28], subsequently in *Croton caudatus* [29], *Croton sphaerogynus* [30], *Croton polycarpus* [31], *Croton campestris* [6], and finally in *C. urucurana* [9]. This indicates a constant presence of the flavonoid in the genus; therefore, **6** may be useful as a possible phytochemical marker. In addition, this flavonoid meets several requirements to be a chemical marker [32] and its effectiveness as a hypoglycemic agent was demonstrated in several studies [33,34]. For this reason, the rutin (**6**) quantification method in the EWE of *C. guatemalensis* was validated. Epicatechin (**7**) was previously described in *Croton lechleri* [35] and *C. urucurana* [36]. This flavanoid was extensively studied as an anti-inflammatory, antioxidant, anti-cancer agent, and as preventing diabetes, cardiovascular diseases, a neuroprotector, and enhancer of muscle performance [37]. Recently, the combination of **7** with rutin (**6**) (75:25) was tested in the oral administration of alloxan-induced hyperglycemic mice for 28 days, and its chronic hypoglycemic activity yielded similar results to glibenclamide [38]. Quercetin (**8**) was previously isolated from *Croton sylvaticus* and proved to be a potent inhibitor of acetylcholinesterase [39]. Quercetin (**8**) was also identified and, in some cases, quantified in *C. sphaerogynus* [30], *C. polycarpus* [31], and *C. urucurana* [9]. This flavonol was extensively studied as an antioxidant, antimicrobial, anti-Alzheimer’s, antiarthritic, anticarcinogenic, and hypoglycemic agent [40]. As far as we know, our current work is the first report of isolation of compounds **1**–**8** from *C. guatemalensis*, and **2** and **3** are new for the genus. 

### 2.3. Quantification of Rutin (**6**) in C. guatemalensis Extract

A comprehensive HPLC method was developed and validated for quantifying rutin (**6**) according to the International Conference on Harmonization guidelines [41]. Rutin (**6**) was selected as a chemical marker based on its constant presence in the genus, stability, and pharmacological activity, as previously mentioned. Diterpenes **3**, **4,** and **5** were not included in the validation process due to their instability. Flavonoids **7** and **8** were neither considered in the validation process due to the lack of a standard for compound **7** and the low concentration in the chromatographic profile in the case of compound **8.** The calibration curve showed good linearity within the test range (R^2^ ≥ 0.9996). The LOD and LOQ values were 0.19 and 0.57 μg/mL, respectively. Intraday and interday precision relative standard deviations (RSDs) were no more than 0.79% (Table 2, Table S1, and Figure S23). No significant degradation of **6** was detected in samples investigated over 72 h at room temperature (20 °C), at 37 °C, and at 4 °C, compared with the initial values. The method was linear, precise, and accurate for the quantitative evaluation of the marker. The content of rutin (**6**) in three batches of *C. guatemalensis* from different years (2014, 2015, and 2019) was investigated and the results are summarized in Table 3. Rutin (**6**) was identified in all batches with amounts between 0.55 and 0.64 mg/g (mg of **6**/g of plant). Previous analyses reported a total of 6.02 mg/g of rutin (**6**) in leaves of a hydroalcoholic extract of *C. campestris* [6], which suggests a possible higher amount of the flavonoid in the leaves or the use of another solvent, such as methanol, as was shown in other studies [42,43].

### 2.4. Affinity-Directed Fractionation

**Figure 6:**
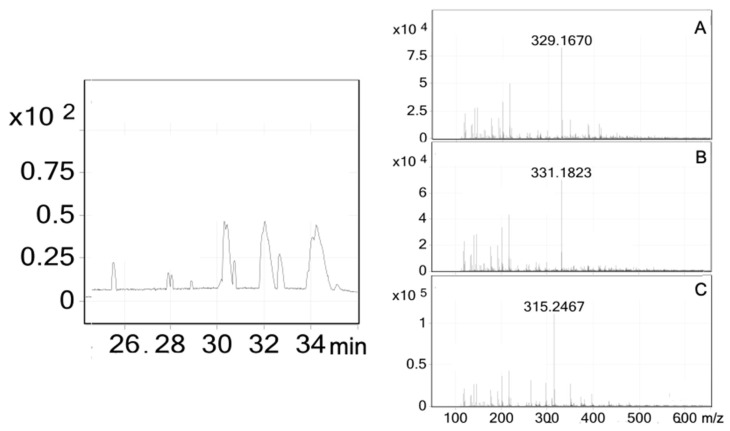
HPLC-MS spectrum obtained from the affinity screening analysis of an EWE soluble extract with *α*-glucosidase: (**A**) the free ligand (*m*/*z* 329.1670 [M–H]^–^; 27.98–28.09); (**B**) the free ligand (*m*/*z* 331.1823 [M–H]^–^; 28.84–28.94 min), and (**C**) the free ligand (*m*/*z* 315.2467 [M–H]^–^; 31.80–32.40 min).

In 2019, the in vivo hypoglycemic effect of the hydroalcoholic and aqueous extracts of *C. guatemalensis* was demonstrated, and the in vitro inhibition of *α*-gucosidases was also tested [17]; this assay did not show inhibition of α-glucosidases from rat intestine, thus, ruling out its hypoglycemic action mechanism as an α-glucosidase inhibitor. However, the extract showed greater activity (IC_50_ = 32 μg/mL) than acarbose (IC_50_ = 105 μg/mL) against α-glucosidases from *Saccharomyces cerevisiae*. The affinity-directed fractionation assay was implemented to find the metabolites responsible for this activity. EWE of *C. guatemalensis* was subjected to a gel permeation chromatography with a spin column packed with polyacrylamide, previously incubated with the α-glucosidases enzymes. The principle of affinity screening is based on the fact that target enzymes incubated with a complex matrix of natural compounds will retain the most tightly non-covalent binding active molecules from a mixture of closely related compounds [44,45]. The HPLC-MS chromatogram obtained from the affinity screening analysis of the EWE allowed the identifying of some of the *ent*-clerodane diterpenes observed in the previous fractionation procedures. Figure 6 illustrates the HRESI-MS obtained from these affinity screening assays: the HRESI-MS obtained from the free ligand (329.1670 [M–H]^–^; 27.98–28.09 min) showed compound **4** (Figure 6a; Table 4), (*m*/*z* 331.1823 [M–H]^–^; 28.84–28.94 min) showed compounds **3** or **5** (Figure 6b; Table 4), and the free ligand (*m*/*z* 315.2467 [M–H]^–^; 30.64–30.79 min) showed compound **1** (Figure 6c; Table 4). Other signals observed in the HPLC-MS spectrum (Figures S24 and S25) are related to other high affinity compounds not observed in the previous fractionation procedures. However, the *m*/*z* yields molecular weights of structures with the same base skeleton (clerodane diterpenes) with one or more oxidations, for example: at 25.47–25.65 min the *m*/*z* is 363.1715, indicating a possible molecular formula, C_21_H_32_O_5_, whereas the peak at 27.84–27.98 min with *m*/*z* 347.1770 could be C_20_H_28_O_5_. Further analysis should be performed to confirm these possible structures. New prototypes of modulatory enzymes observed with affinity studies allowed knowing that these diterpenes had a high affinity for the *S. cerevisiae α*-glucosidase enzyme.

The importance of these experiments could be explained by the hypothesis of Kimura [46] that yeast and mammalian α-glucosidases belonged to two different families that differed in their amino acid sequences and their abilities to act on different substrates. The yeast and insect enzymes belong to family I (GH13) and have greater affinity for heterogeneous substrates, such as sucrose or 4-PNGP, whereas α-glucosidases from mammals belong to family II (GH31) and have greater affinity for homogeneous substrates, such as maltose.

In this sense, according to our findings, the inhibition of the Saccharomyces enzymes by the compounds could be more related to an ecological role that enables the plants to defend themselves against insect herbivory or fungal attacks by inhibiting type 1 enzymes.

### 2.5. Molecular Docking

**Figure 7:**
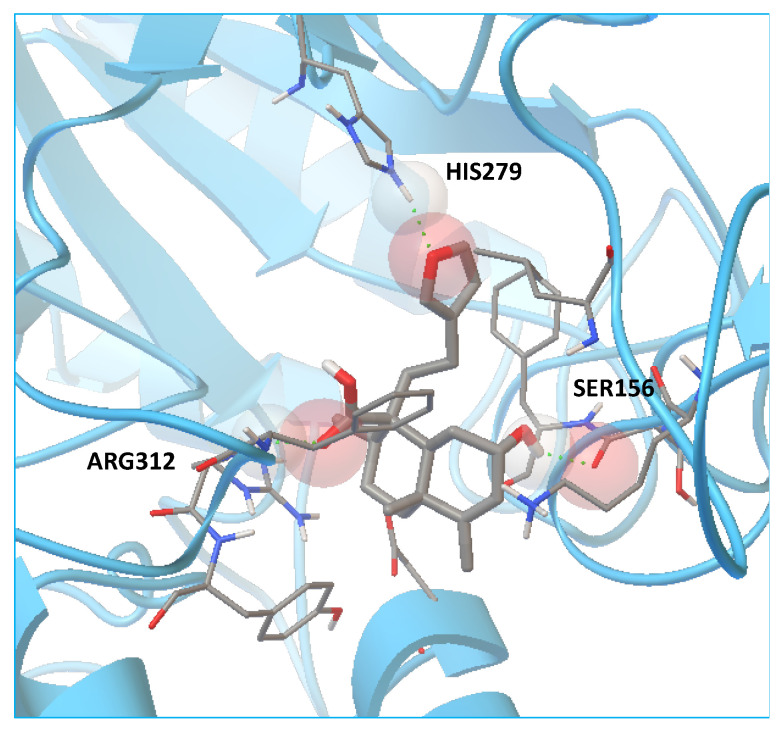
Docking results of the enzyme binding conformation of compound **5** with MAL12, three hydrogen bonds formed with catalytic residues SER156, HIS279, and ARG312.

Compounds **1**–**5** and acarbose (control) were constructed in 3D models and molecular docking studies between ligands (acarbose and compounds **1**–**5**) and the amino acid sequence of *α*-glucosidase from *S. cerevisiae* (MAL12) and human maltase-glucoamylase (MGAM-C) by AutoDock 4.2 software were performed to improve our understanding of the interaction of the high affinity compounds **1–5** inside the catalytic sites of MAL12 and MGAM-C, which were selected as the template for molecular modeling to establish a comparison between the resulting affinity-directed fractionation assay and the theoretical inhibition constant (*K*i) obtained from in silico studies. To refine the results, the best conformations observed in the preliminary analysis were docked into a smaller area of the catalytic domain. Data are shown in Table 4. Acarbose fits well in the catalytic pocket of the analyzed enzymes and showed hydrogen-bonding interactions with the amino acid residues HIS279 (2.08 Å), GLN322 (2.00 Å), and ARG312 (2.15 Å) with MAL12, whereas the binding modes inside the catalytic site of MGAM-C corresponded to TYR1251 (1.93 Å), GLN1372 (1.75 Å), ARG1377 (2.11 Å), GLN1561 (2.07 Å), and GLY1588 (2.17 Å). Compounds **1**–**5** fit well in the catalytic pocket with MAL12 and showed hydrogen-bonding interactions with the amino acid residues HIS279 and ARG312, and preserved catalytic residues around TYR1251 in MGAM, which is involved in the catalytic substrate specificity of this protein [47]. Compounds **4** and **5** have the lowest *K*i values of both analyzed enzymes (Table 4). These results plus the results of affinity studies with *α*-glucosidase indicate that the best conformation for enzyme inhibition is that of compound **5** (Figure 7).

**Figure 8:**
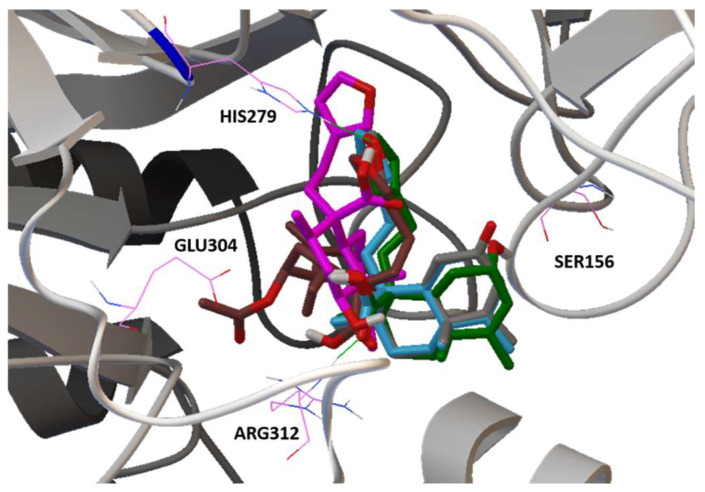
Pharmacophore docking results show the minimized structure of MAL12 with the active compounds **1**–**5**, the furane group forms a hydrogen bond with the NH of the catalytic residue HIS279.

A secondary study was carried out in the catalytic site, using acarbose as a control and the best conformation of each compound **1**–**5** of the refine study. This allowed knowing the pharmacophore of compounds **1**–**5**. Figure 8 shows the minimized structure of the *α*-glucosidase complexed with active compounds **1**–**5** in the hypothesized binding mode. The furane group at C-13 of all compounds forms a hydrogen bond with the NH of the catalytic residue HIS279, inducing a greater steric impediment at the surface of the catalytic pocket.

## 3. Conclusions

In this study, eight compounds were isolated from de bark of *C. guatemalensis*, and the absolute configuration of two unreported *ent*-clerodane diterpenoids (**2** and **3**) were established by ECD spectrum. Quantification of the flavonoid rutin (**6**) was validated and analysis of three different batches indicated very similar amounts of rutin (**6**) content in each of them.

The approach for affinity-directed fractionation was applied at various stages during the isolation and purification processes to speed the identification of new *α*-glucosidase inhibitors, which could have an impact in the microscale separation and dereplication of active natural products, as demonstrated here for the clerodanes from *C. guatemalensis.* The present study provides insights into the phytochemical composition of the hydroalcoholic extract of *C. guatemalensis* and reveals new prototypes of enzyme modulators through affinity studies. As previously mentioned, these findings could be related to an ecological role that enables the plant to defend themselves against herbivory insects or fungal attacks by inhibiting enzymes of family I, according to Kimura [46]. Because, in the present work, we used similar extracts to those previously tested [17], some of the compounds isolated herein could be involved in the previously observed hypoglycemic activity. However, more experiments are needed to confirm this.

## 4. Materials and Methods

### 4.1. General Experimental Procedure

Analytical and preparative HPLC analyses were performed in an Agilent 1260 Infinity system equipped with a G1311B quaternary pump, G1367E autosampler, G1315C DAD VL+, and controlled by Agilent ChemStation software (Agilent Technologies, Inc., Santa Clara, CA, USA). For analytical and semipreparative HPLC, a Luna Omega Polar C_18_, 50 × 2.1 mm id., 1.6 μm column (Phenomenex, Inc., Torrance, CA, USA) was used. Rutin and quercetin standards (>94% HPLC) were purchased from Sigma-Aldrich (St. Louis, MO, USA). Column chromatography (CC) was carried out on silica gel (70–230 mesh, Merck Mexico) or Sephadex LH-20 (Sigma-Aldrich Chemical). Thin-layer chromatography analyses were carried out on silica gel 60 F_254_ plates (Macherey-Nagel, Düren, Germany) using ceric sulfate (10%) solution in H_2_SO_4_ as color reagent. NMR spectra including ^1^H, ^13^C, DEPT, HSQC, HMBC, COSY, NOESY, and TOCSY were recorded in a Varian Inova spectrometer (Varian, Inc., Palo Alto, CA, USA) at 400 (^1^H) and 95 MHz (^13^C) or Bruker DMX500 spectrometer (Bruker Nano GmbH, Berlin, Germany) operating at 500 MHz (^1^H) or 125 MHz (^13^C) NMR; chemical shifts were recorded as δ values. High-resolution ESI-MS was measured in a coupled liquid chromatography system with single quadruple mass spectrometry and time of flight (HPLC-EM-SQ-TOF Model G6530BA, Agilent Technologies, Inc.). HR-MS data were obtained using a Jeol, AccuTOF JMS-T100LC mass spectrometer (HR-DART-MS) (JEOL USA, Peabody, MA, USA). ECD data were obtained using a JASCO, J-1500 CD spectrometer (JASCO, Oklahoma City, OK, USA). IR data were obtained using a FT-IR Bruker Tensor 27 spectrometer.

### 4.2. Plant Material and Extracts

*Croton guatemalensis* was collected by Dr. Carola Cruz, based on previous ethnobotanical studies (Cruz, 2011), at the Department of Chimaltenango, Guatemala, in 2019.

Ethanol–water extract (EWE) was made by heating 20 g of the dry plant material with a mixture of ethanol:water (1:1; 500 mL) during 2 h, followed by filtration and concentration under reduced pressure to remove ethanol in a rotary vacuum evaporator (Büchi Labortechnick, AG, Flawil, Switzerland) at 40 °C. Finally, it underwent lyophilization to yield 4.058 g of EWE. The extract was stored at 4 °C for HPLC analysis.

For phytochemical analysis, the dried and ground material (60 g) of *C. guatemalensis* was extracted with a mixture of ethanol:water (1500 mL) during two hours and then filtered and extracted with CH_2_Cl_2_ (3 × 1500), followed by extraction with ethyl acetate (3 × 1500), to yield 3.7525 g of CH_2_Cl_2_-soluble fraction (DSF), 754 mg of EtOAc-soluble fraction (ESF), and 5.2820 g of H_2_O-soluble fraction (WSF).

### 4.3. Isolation Compounds

DSF (3.70 g) was partitioned by column chromatography (CC) on 95 g of silica gel (70–230 mesh, Merck Mexico) using mixtures of *n*-hexane/EtOAc/MeOH as eluent, starting with *n*-hexane 100%, increasing the polarity with EtOAc until 100%, and subsequently with MeOH, obtaining 164 collections of 50 mL that were gathered according to their chromatographic profile analyzed by TLC. This process led to 35 primary fractions (DSF1- DSF35). Fraction DSF1 (1.16 g) was obtained as the pure compound **1**; preparative TLC of fractions DSF6 (122.3 mg; CH_2_Cl_2_:MeOH, 97:3; 2.0 mm), DSF11 (58.3 mg; *n*-hexane:EtOAc, 75:25, 1.0 mm), and DSF13 (62.1 mg; *n*-hexane:EtOAc:Me_2_CO; 50:45:5; 1 mm) yielded 64.0 mg of **2**, 16.0 mg of **3,** and 16.6 mg of **4**; 54.5 mg of DSF14 was subjected to TLC (CH_2_CL_2_:MeOH; 97:3) to obtain 10.5 mg of **5,** and 7.5 mg of **4**.

ESF (692 mg) was subjected to Sephadex LH-20 using MeOH 100% as eluent, and this process led to 20 subfractions (ESF1-ESF20); ESF8 (15.2 mg) was analyzed by HPLC to be compared with the UV spectrum of a standard of rutin (**6**) (>94% HPLC; Sigma-Aldrich) and was corroborated by its mass spectrum (ESI-MS). ESF10 (37.0 mg) was resolved by semi-preparative HPLC (Nucleosil 250 × 10 mm i.d., 5 μm, C18 Macherey-Nagel), using a mixture of 15:85 MeCN:H_2_O as mobile phase during 15 min (2.0 mL/min; 280 nm UV-det.) to obtain 8.2 mg of **7** (Rt = 12.5 min).

MeOH (200 mL) was added to the WSF to obtain 672 mg soluble in methanol, which was subjected to Sephadex LH-20 using MeOH 100% as eluent. This process led to 17 subfractions (WSF1-WSF17); WSF16 (9.1 mg) was isolated as the pure compound **8** and analyzed by HPLC to be compared with the UV spectrum of a quercetin standard (>94% HPLC; Sigma-Aldrich), which was confirmed.

#### 4.3.1. 6(*s*)-Acetoxy-15,16-diepoxy-*ent*-cleroda-3,13(16),14-trien-20-oic Acid (Crotoguatenoic Acid A; **2**)

White powder; [α]_D_^20^-12.0 (c 0.001 MeOH); UV (MeOH) λ_max_ (log ε) 210 (0.972) nm; ECD (MeOH) λ_max_ (Δε) 204 (−2.73), 236 (+4.40); IR νmax 3380 (OH), 1714 (COOH), 1693 (C=O), 1408 (C=C), 1358 (C=C), 1372 (C=C) cm^−1^; ^1^H (CDCl_3_, 400 MHz), and ^13^C NMR (CDCl_3_, 150 MHz), see Table 1; ESI-MS 397.4 [M + Na]^+^ and HRESIMS 373.1813 [M–H]^−^ (calcd. for C_22_H_29_O_5_, 373.2020).

#### 4.3.2. 6(*s*)-Hydroxy-15,16-diepoxy-*ent*-cleroda-3,13(16),14-trien-20-oic Acid (Crotoguatenoic Acid B; **3**)

White powder; [α]_D_^20^-12.3 (c 0.001 MeOH); UV (MeOH) λ_max_ (log ε) 234 (1.34); ECD (MeOH) (Δε) 204 (−0.3.36), 232 (+5.12); IR νmax 3399 (OH), 1695 (C=O),1450 (C=C), 1376 (C=C) cm^−1^; ^1^H (CDCl_3_, 400 MHz) and ^13^C NMR (CDCl_3_, 150 MHz), see Table 1; HRESIMS *m*/*z* 333.20515 [M + H]^+^ (calcd. for C_20_H_29_O_4_, 333.20658).

### 4.4. HPLC Analysis

High performance liquid chromatography (HPLC) was developed using an Agilent 1260 HPLC instrument equipped with an Agilent G1315C UV diode array detector (DAD). Chromatographic profile elaboration was performed using a Phenomenex (Luna Omega Polar C_18_, 50 × 2.1 mm id., 1.6 μm) reverse phase column. Elution was carried out at a flow rate of 0.35 mL/min with water as solvent A, containing 0.1% formic acid and acetonitrile (MeCN) as solvent B, starting with a gradient elution of 99:1 (A:B), 80:20 (A:B) at 14 min, 50:50 (A:B) at 14–26 min, 70:30 (A:B) at 26–34 min, 20:80 (A:B) at 34–35 min, and 99:1 (A:B) at 35–38 min. The column temperature was kept at 35 °C. System control, data collection, and processing were accomplished using the OpenLAB LC 1260 chromatography software. Working solutions of samples (EWE, fractions, and isolated compounds) of *C. guatemalensis* were prepared by dissolving 10.0 mg of EWE in 1 mL of a mixture of MeCN:H_2_O (1:1) or 1 mg of the compound in 1 mL of the required solvent according to its solubility (EtOH, MeOH, MeCN, or H_2_O), which were injected (2 μL) using an autosampler. For UV detection, the wavelength program was set at an acquisition of λ 205, 240, 254, 280, and 365 nm.

### 4.5. HPLC Method Validation

The method was validated according to the ICH guidelines for specificity, linearity, accuracy, precision, LOQ, and LOD [41]. Specificity was checked using the extract and a rutin (**6**) standard. Linearity of the method was evaluated by inspection of a rutin (**6**) standard solution at a concentration range of 20 to 250 µg/mL. A calibration line was made, and the least square line and correlation coefficient were calculated. Accuracy was evaluated by means of recovery assays carried out by adding known amounts of the standards of **6** to the sample at three different levels of the initial concentration of the sample. Average recoveries were calculated by the Equation (1).

(1) $$Recovery\;\left(\%\right)=\frac{\left(amount\;found-original\;amount\right)}{amount\;spiked}\times 100$$

Precision was evaluated by repeatability using six replicates at 100% of the test concentration. Stability was tested by analyzing the sample solution at different time points (0, 24, 48, and 72 h). LOD and LOQ were quantified based on the standard deviation (σ) of the response and the slope (S) calculated by the equations 2 and 3, respectively.

(2) $$\mathrm{LOD}=\frac{3.3\sigma }{S}$$

(3) $$\mathrm{LOQ}=\frac{10\sigma }{S}$$

### 4.6. Affinity-Directed Fractionation

Gel permeation chromatography was performed with a spin column (BioRad Laboratories, Hercules, CA, USA) packed with polyacrylamide, 1 cm high, 100 μL swollen). The gel and samples were prepared in a solution of 0.1 M sodium phosphate buffer (pH 6.8) [45]. Aliquots (10 μL; in triplicates) of the extract (200 μg/mL) and acarbose (therapeutic control) were independently incubated for 5 min with 20 μL of the enzyme stock solution (0.9 units/mL of yeast *α*-glucosidase in 100 μM of buffer solution). Upon loading the test samples at the top of the spin exclusion column, the mixtures were eluted by centrifugation at RCF 42,985 *g* for 4 min; then, the eluate, corresponding to the solvent front and containing the *α*-glucosidase-acarbose complex, was collected and a denaturing solution (10 μL) of 3% glacial acetic acid in acetonitrile:water (1:1, v:v) was added and mixed with a vortex mixer. The solution was vacuum-dried and reconstituted with acetonitrile and analyzed by a coupled liquid chromatography system with single quadruple mass spectrometry and time of flight (HPLC-EM-SQ-TOF). Chromatographic profile elaboration was performed using a Phenomenex (Kinetex C_18_, 50 × 2.1 mm id., 2.6 μm) reverse phase column; the same flow gradient conditions mentioned above (item 4.4) were used. ESI mass spectra after the SEC/ESI-MS protocol for the acetonitrile and the enzyme functioned as background signals for the spectrum of the samples of interest.

### 4.7. Molecular Docking

Docking was carried out with the AutoDock 4.2 software (The Scripps Research Institute, La Jolla, CA, USA) using the default parameters. The molecular docking was performed with a model built by homology with *Bacillus cereus α*-glucosidase (1UOK.PDB) for the amino acid sequence of MAL12 from *S. cerevisiae*, which was retrieved from the UniProt protein resource data bank (accession code P5334) with preserved catalytic residues His111, Asp205, Glu276, His348, and Asp349 [48]. All files were prepared by adding polar hydrogen atoms and merged non-polar hydrogens to the enzyme structures and computing Gasteiger charges for the molecular model of analyzed compounds (**1**–**5**) as previously described for acarbose [48]. The entire system was subjected to a surface scanning and refined docking.

### 4.8. Computational Details

The Spartan’14 software was implemented to calculate the energy-minimized form with geometric optimization for all ligands, utilizing a semiempirical method (PM3). The resulting conformers were filtered and checked for redundancy. All conformers were minimized using a DFT force field at the B3LYP/DGDZVP level of theory employing Gaussian 09 software. The conformers were optimized, and thermochemical properties, IR, and vibrational analyses were obtained at the same level of theory. The TD-SCF with the default solvent model was used to perform the theoretical circular dichroism (TCD) calculations of the major conformers in the MeOH solution, using a B3LYP/DGDZVP force field. The calculated excitation energy (nm) and rotatory strength (R) in dipole velocity (R_vel_) form was simulated into a TCD curve using the Harada–Nakanishi equation, as implemented in the SpecDis 1.71 software [49].

## Figures and Tables

**Table 1:** ^1^H and ^13^C NMR spectroscopy data of compounds **2** and **3** (*δ* in ppm, *J* in Hz).

	2 ^a^		3 ^a^	
Position	*δ*_H_	*δ*_C_	*δ*_H_	*δ*_C_
**1**	*α* 1.85 ^b^, m *β* 1.93 ^b^, m	19.8	*α* 1.84 ^b^, m *β* 1.93 ^b^, m	20.1
**2**	*α* 1.99 ^b^, m *β* 2.07 ^b^, m	27.1	*α* 2.02 ^b^, m *β* 2.10 ^b^, m	27.2
**3**	5.30, dd (2.87, 1.39)	123.6	5.31, m	123.1
**4**	-	141.5	-	143.0
**5**	-	42.9	-	44.7
**6**	4.73 dd (11.34, 5.00)	78.2	3.59, dd (11.35, 4.97)	76.1
**7**	*α* 2.20 ^b^, m *β* 1.76 ^b^, m	33.0	*α* 2.20 ^b^, dd (13.71, 2.26) *β* 1.70 ^b^, m	37.4
**8**	1.76 ^b^, m	34.3	1.71 ddd (14.06, 6.21, 3.58)	34.7
**9**	-	49.3	-	49.4
**10**	1.66, d (11.50)	47.1	1.58 dd (11.63, 1.30)	47.2
**11**	*α* 1.85 ^b^, m *β* 2.26 ^b^, m	33.9	*α* 1.93, m *β* 2.26 ^b^, m	34.0
**12**	2.33 ^b,c^, m	17.8	2.33 ^b,c^, m	17.8
**13**	-	124.4	-	124.5
**14**	6.26, dd (1.83, 0.90)	110.9	6.26, dd (1.83, 0.95)	110.9
**15**	7.35, t (1.69)	143.1	7.35, t (1.69)	143.0
**16**	7.23, dd (1.61, 0.88)	138.7	7.23, dd (1.62, 0.91)	138.7
**17**	1.14, d (6.69)	16.1	1.15, d (6.71)	16.3
**18**	1.59, br s	21.2	1.84, br s	22.6
**19**	1.09, s	13.9	0.98, s	12.8
**20**	-	182.3	-	181.9
**-OAc**	2.04	22.0	-	-
		170.8		

^a^ Data recorded at 400 MHz (^1^H) and 150 MHz (^13^C) in CDCl_3._^b^ Overlapped signals. ^c^ Signals for two protons.

**Table 2:** Validation report of the method for rutin (**6**) determination in *C. guatemalensis*.

*R*_t_	Linear Range (μg/mL)	Calibration Equation	*R* ^2^ ^ a^	LOD (μg/mL)	LOQ (μg/mL)	Precision		Recovery (%mean)
						Intraday (%RSD)	Interday (%RSD)	
14.52	20–250	*Y* = 9.29484284*x* + 17.083753	0.9996	0.19	0.57	0.79	0.22	100.74

^a^*R*^2^ correlation coefficient for five data points in the calibration curves (*n* = 3).

**Table 3:** Content of rutin (**6**) in *C. guatemalensis*.

Batch	%EWE ^a^	Content in mg/g ^b^
09–2013	20.5	0.6067 ± 0.0025
06–2015	19.3	0.5585 ± 0.0042
10–2019	18.7	0.6440 ± 0.0068

^a^ Yield in grams of extract per grams of plant material; ^b^ (mg of **6**/g of plant material); data are mean ± SD; *n* = 3.

**Table 4:** Clerodanes identification with *α*-glucosidase enzyme and docking studies.

Compound	Formula ^a^	ESI-MS [M–H]^– b^	ESI-MS [M + H]^+ b^	MAL12		MGAM	
				Theoretical Ki	Hydrogen Bond	Theoretical Ki	Hydrogen Bond
**1**	C_20_H_28_O_3_ (316)	315.2467	-	7.12 μM	His279, Arg312	3.02 μM	Gln1372, Arg1377
**2**	C_22_H_30_O_5_ (374)	-	-	17.1 μM	His279, Arg312	6.31 μM	Gln1372, Arg1377
**3**	C_20_H_28_O_4_ (332)	331.1823	333.2444	13 μM	Arg312	5.09 μM	Gln1372, Arg1377
**4**	C_20_H_26_O_4_ (330)	329.1670	-	6.73 μM	His279, Arg312	1.95 μM	Gln1372, Arg1377
**5**	C_20_H_28_O_4_ (332)	331.1823	333.2444	4.14 μM	Ser156, His279, Arg312	2.4 μM	Try1251, Gln1372, Arg1377
Acarbose ^c^	C_25_H_43_NO_18_ (645)	-	646	51.4 nM	His279, Gln322, Glu304, Arg312	35.7 nM	Tyr1251, Gln1372, Arg1377, Gln1561, Gly1588

^a^ Molecular weight (Da). ^b^ Observed in affinity studies. ^c^ Positive control substance.

## Data Availability

All data in this study can be found in the manuscript or in the Supplementary Materials.

## Supplementary Materials

The following supporting information can be downloaded at: https://www.mdpi.com/article/10.3390/plants11223159/s1. Figure S1: The ^1^H NMR spectrum of **2** in CDCL_3_ (500 MHz); Figure S2: The ^13^C NMR spectrum of **2** in CDCL_3_ (125 MHz); Figure S3: The DEPT spectrum of **2** in CDCL_3_ (125 MHz); Figure S4: The HSQC spectrum of **2** in CDCL_3_; Figure S5: The ^1^H-^1^H COSY spectrum of **2** in CDCL_3_; Figure S6: The HMBC spectrum of **2** in CDCL_3_; Figure S7: The NOESY spectrum of **2** in CDCL_3_; Figure S8: The TOCSY spectrum of **2** in CDCL_3_; Figure S9: The ESIMS spectrum of compound **2**; Figure S10: The UV spectrum of compound **2**; Figure S11: The IR spectrum of compound **2**; Figure S12: The ^1^H NMR spectrum of **3** in CDCL_3_ (500 MHz); Figure S13: The ^13^C NMR spectrum of **3** in CDCL_3_ (125 MHz); Figure S14: The DEPT spectrum of **3** in CDCL_3_ (125 MHz); Figure S15: The HSQC spectrum of **3** in CDCL_3_; Figure S16: The ^1^H-^1^H COSY spectrum of **3** in CDCL_3_; Figure S17: The HMBC spectrum of **3** in CDCL_3_; Figure S18: The NOESY spectrum of **3** in CDCL_3_; Figure S19: The TOCSY spectrum of **3** in CDCL_3_; Figure S20: The HRESIMS spectrum of compound **3**; Figure S21: The UV spectrum of compound **3**; Figure S22: The IR spectrum of compound **3**; Figure S23: Calibration curve of rutin (**6**); Figure S24: HPLC-ESIMS spectrum obtained from the affinity screening analysis of the EWE soluble extract with *a*-glucosidase, positive mode; Figure S25: HPLC-ESIMS spectrum obtained from the affinity screening analysis of the EWE soluble extract with *a*-glucosidase, negative mode; Table S1: Standard calibration curve of rutin (**6**).
