# Supplementary material for: Phytochemical Screening and Isolation of New Ent-Clerodane Diterpenoids from Croton guatemalensis Lotsy
Source: Plants (Basel). 2022 Nov 18;11(22):3159. doi: 10.3390/plants11223159 (PMC9692395; doi:10.3390/plants11223159)
Supplement: Supplementary file 1 [file plants-11-03159-s001.zip › plants-1947981-supplementary.pdf]

Phytochemical Screening and Isolation of New *Ent*-Clerodane Diterpenoids from *Croton guatemalensis* Lotsy

Sonia Marlen Escandón-Rivera [1], Adolfo Andrade-Cetto [1,*], Daniel Genaro Rosas-Ramírez [2] and Roberto Arreguín-Espinosa [2]

[1] Departamento de Biología Celular, Facultad de Ciencias, Universidad Nacional Autónoma de México, Av. Universidad 3000, Circuito Exterior S/N, Coyoacán, Ciudad Universitaria, Mexico City 04510, Mexico
[2] Departamento de Biomacromoléculas, Instituto de Química, Universidad Nacional Autónoma de México, Av. Universidad 3000, Circuito Exterior S/N, Coyoacán, Ciudad Universitaria, Mexico City 04510, Mexico
* Correspondence: aac@ciencias.unam.mx; Tel.: +52-5556225437

Table of contents

Figure S1. The ^{1}H NMR spectrum of **2** in $CDCL_3$ (500 MHz).
Figure S2. The ^{13}C NMR spectrum of **2** in $CDCL_3$ (125 MHz).
Figure S3. The DEPT spectrum of **2** in $CDCL_3$ (125 MHz).
Figure S4. The HSQC spectrum of **2** in $CDCL_3$.
Figure S5. The ^{1}H-^{1}H COSY spectrum of **2** in $CDCL_3$.
Figure S6. The HMBC spectrum of **2** in $CDCL_3$.
Figure S7. The NOESY spectrum of **2** in $CDCL_3$.
Figure S8. The TOCSY spectrum of **2** in $CDCL_3$.
Figure S9. The ESIMS spectrum of compound **2**.
Figure S10. The UV spectrum of compound **2**.
Figure S11. The IR spectrum of compound **2**.
Figure S12. The ^{1}H NMR spectrum of **3** in $CDCL_3$ (500 MHz).
Figure S13. The ^{13}C NMR spectrum of **3** in $CDCL_3$ (125 MHz).
Figure S14. The DEPT spectrum of **3** in $CDCL_3$ (125 MHz).
Figure S15. The HSQC spectrum of **3** in $CDCL_3$.
Figure S16. The ^{1}H-^{1}H COSY spectrum of **3** in $CDCL_3$.
Figure S17. The HMBC spectrum of **3** in $CDCL_3$.
Figure S18. The NOESY spectrum of **3** in $CDCL_3$.
Figure S19. The TOCSY spectrum of **3** in $CDCL_3$.
Figure S20. The HRESIMS spectrum of compound **3**.
Figure S21. The UV spectrum of compound **3**.
Figure S22. The IR spectrum of compound **3**.
Figure S23. Calibration curve of rutin (**6**).
Figure S24. HPLC-ESIMS spectrum obtained from the affinity screening analysis of the EWE soluble extract with *α*-glucosidase, positive mode.
Figure S25. HPLC-ESIMS spectrum obtained from the affinity screening analysis of the EWE soluble extract with *α*-glucosidase, negative mode.
Table S1. Standard calibration curve of rutin (**6**).

Figure S1. The ^{1}H NMR spectrum of **2** in CDCL3 (500 MHz).

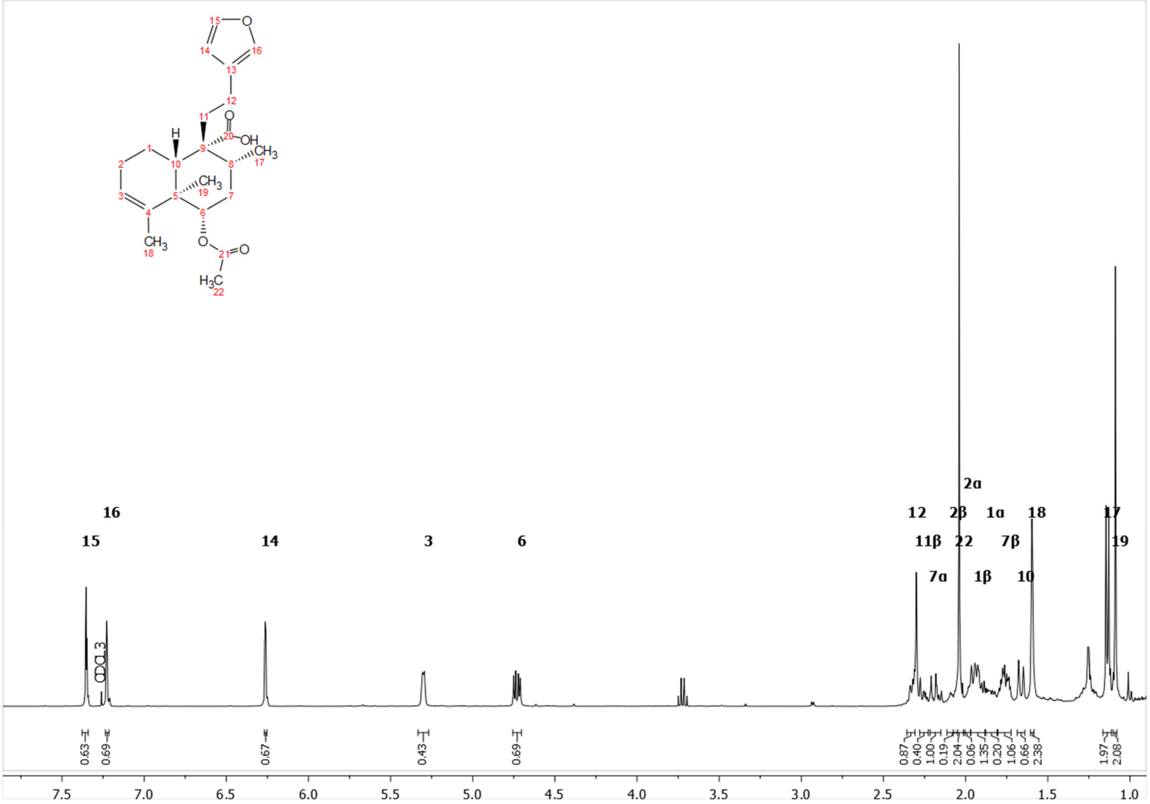

Figure S2. The ^{13}C NMR spectrum of **2** in CDCL3 (125 MHz).

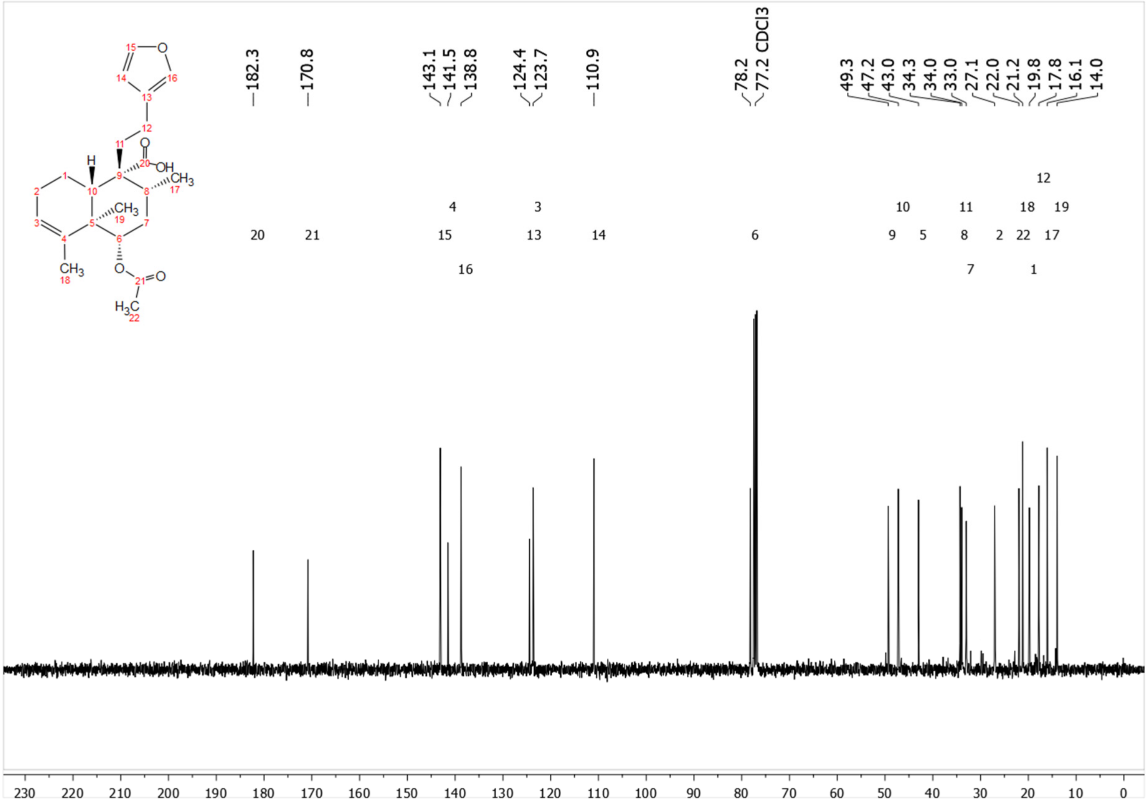

Figure S3. The DEPT spectrum of **2** in $CDCL_3$ (125 MHz).

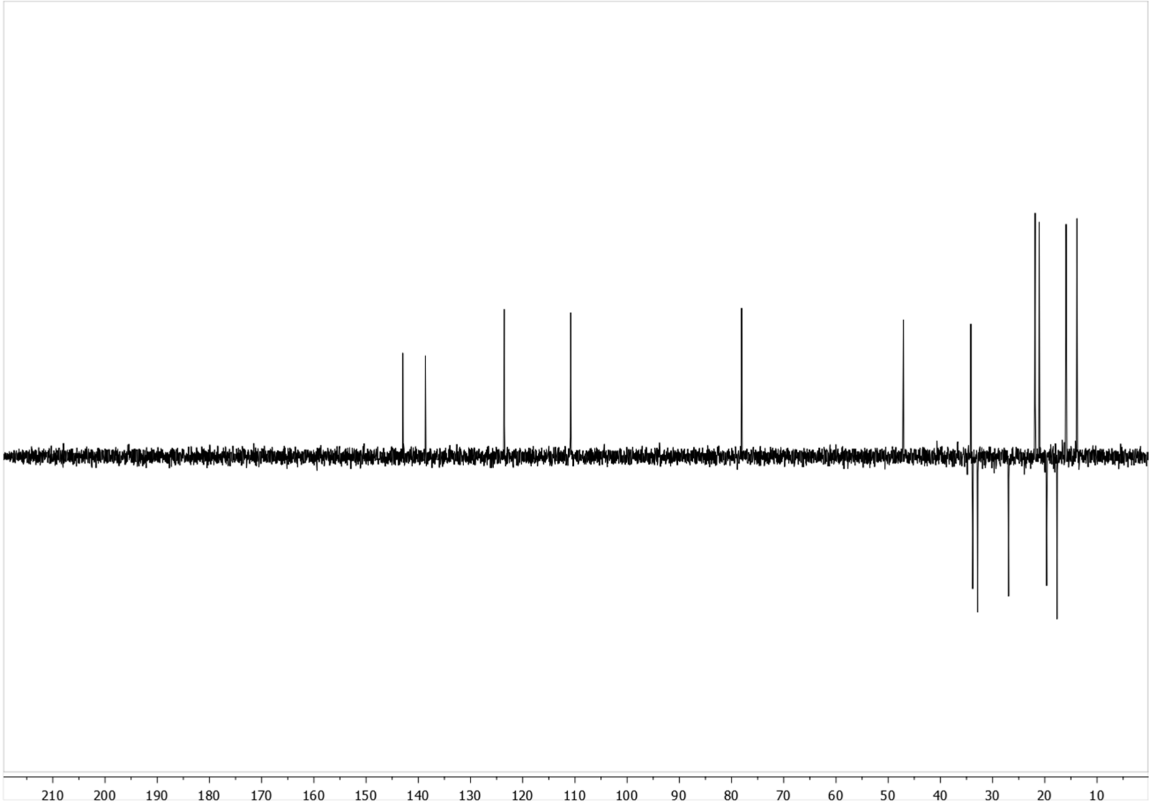

Figure S4. The HSQC spectrum of **2** in $CDCL_3$.

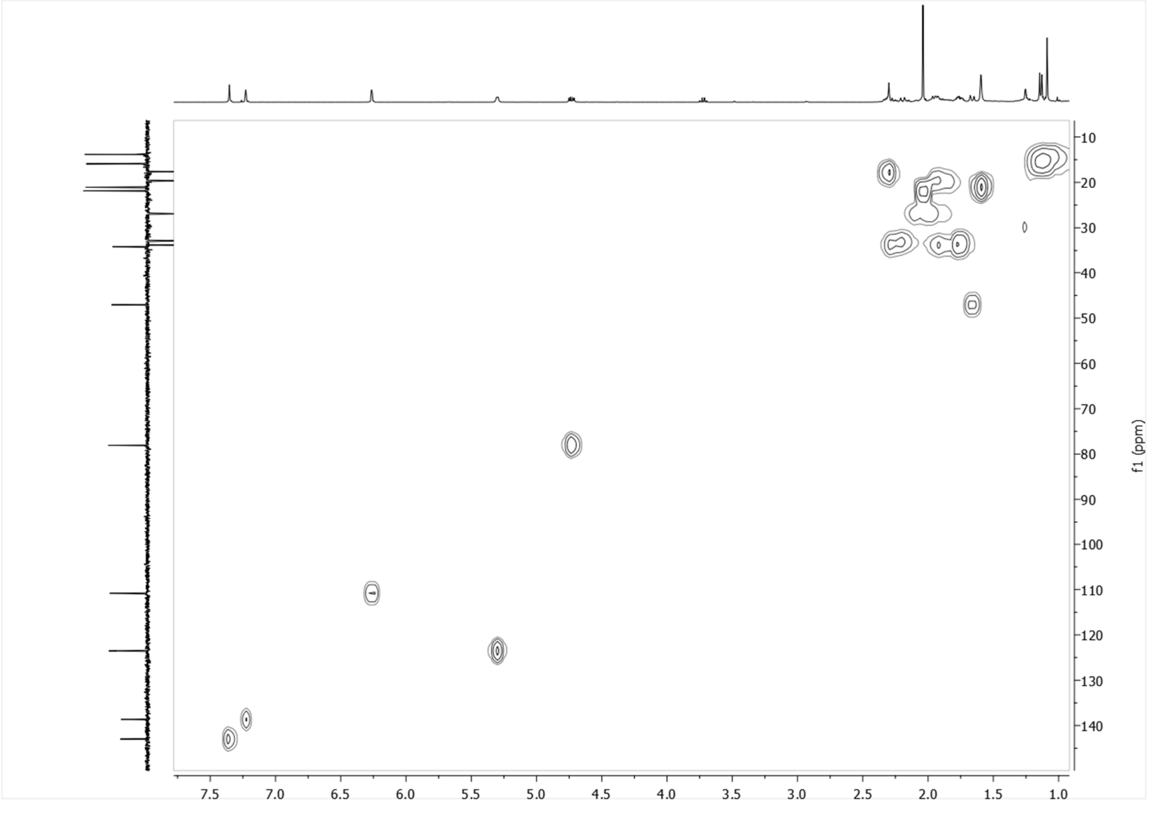

Figure S5. The ^1H-^1H COSY spectrum of **2** in CDCL3.

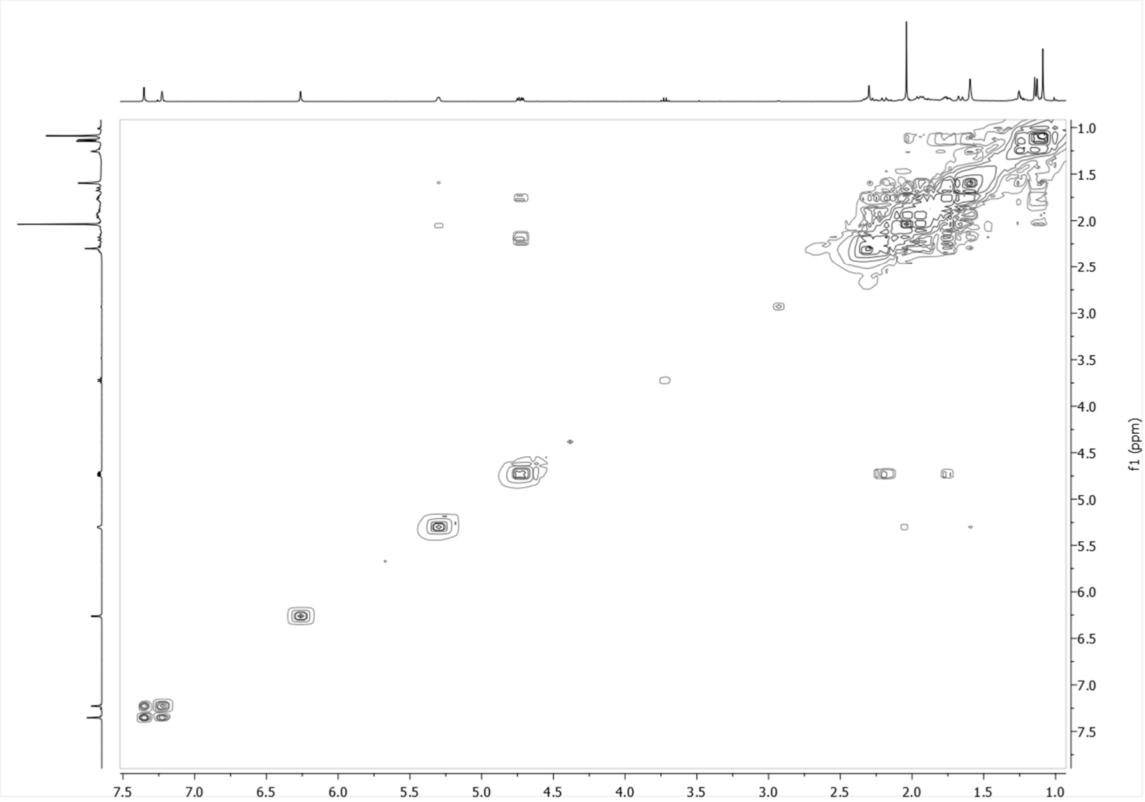

Figure S6. The HMBC spectrum of **2** in CDCL3.

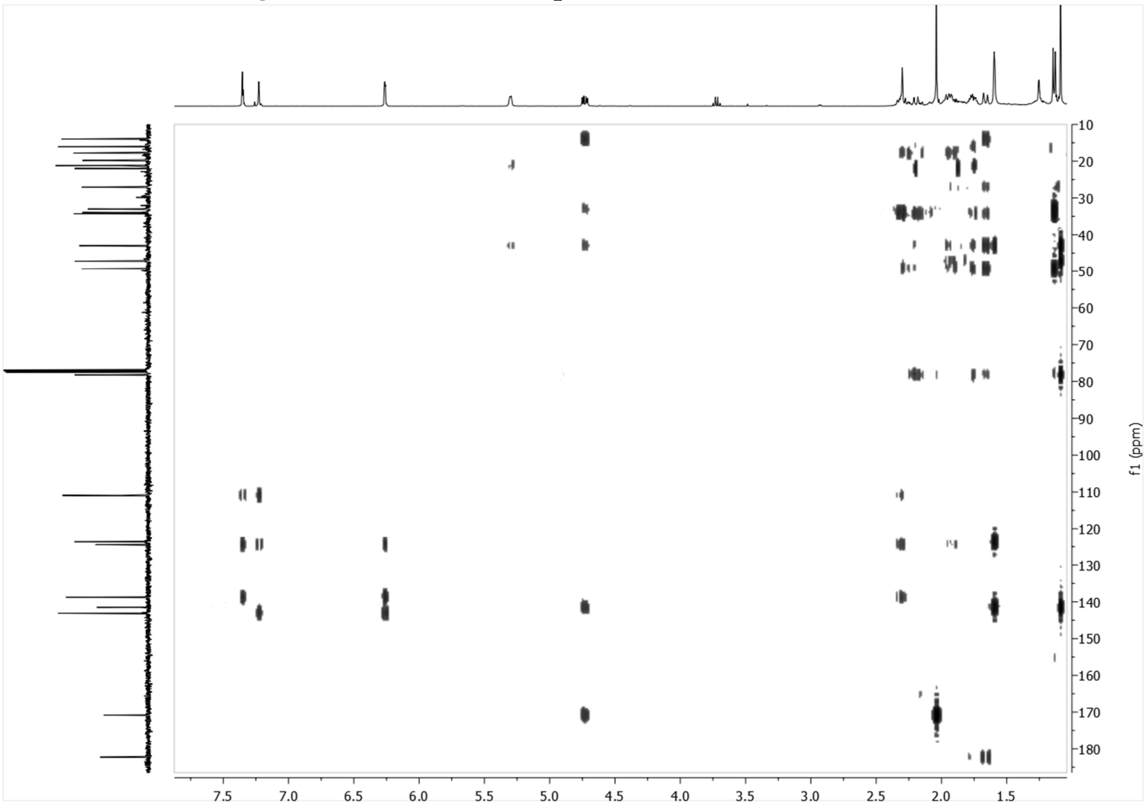

Figure S7. The NOESY spectrum of **2** in CDCL3.

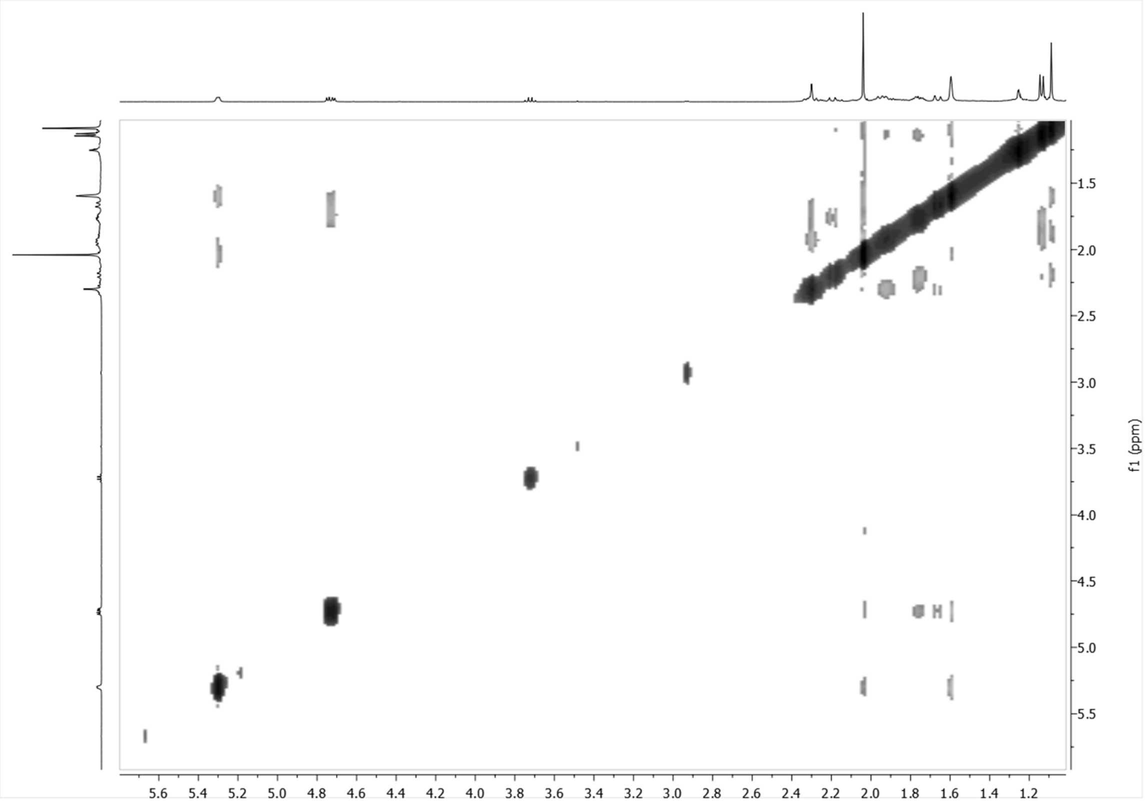

Figure S8. The TOCSY spectrum of **2** in CDCL3.

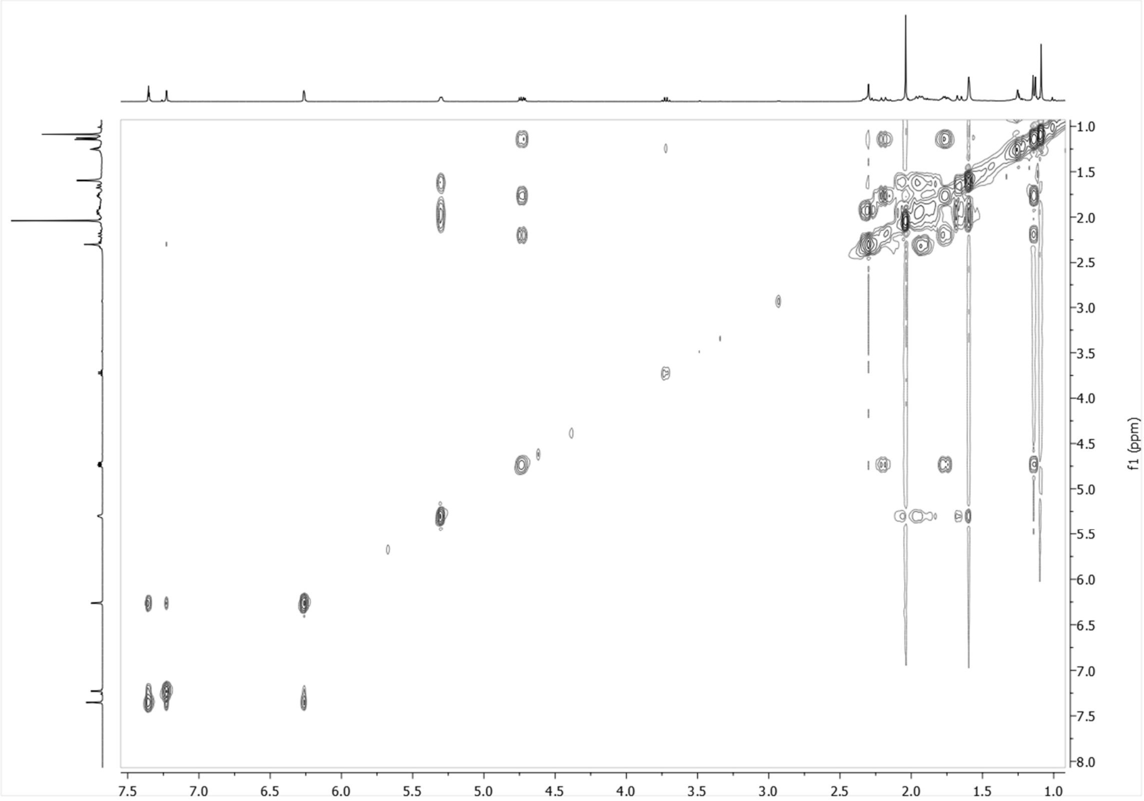

Figure S9. The HRESIMS spectrum of compound **2**.

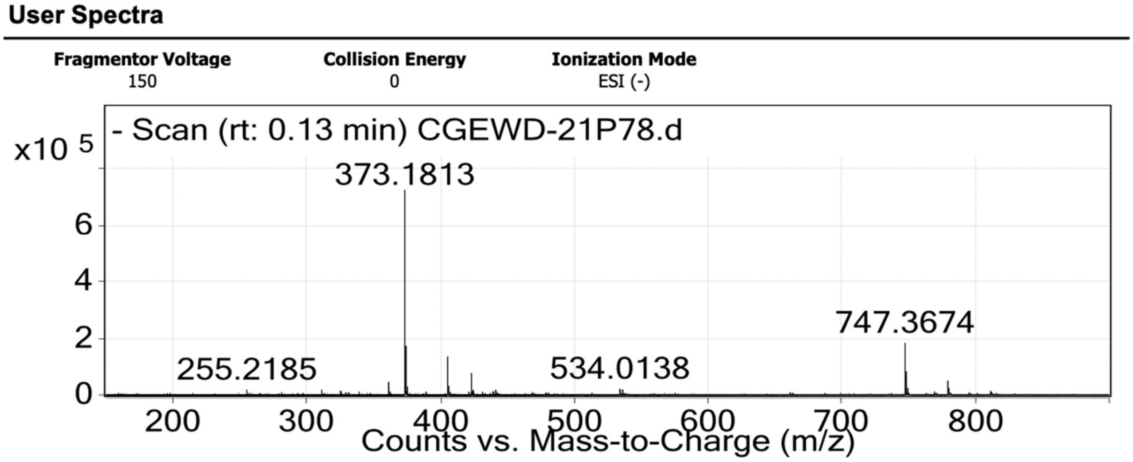

Figure S10. The UV spectrum of compound **2**.

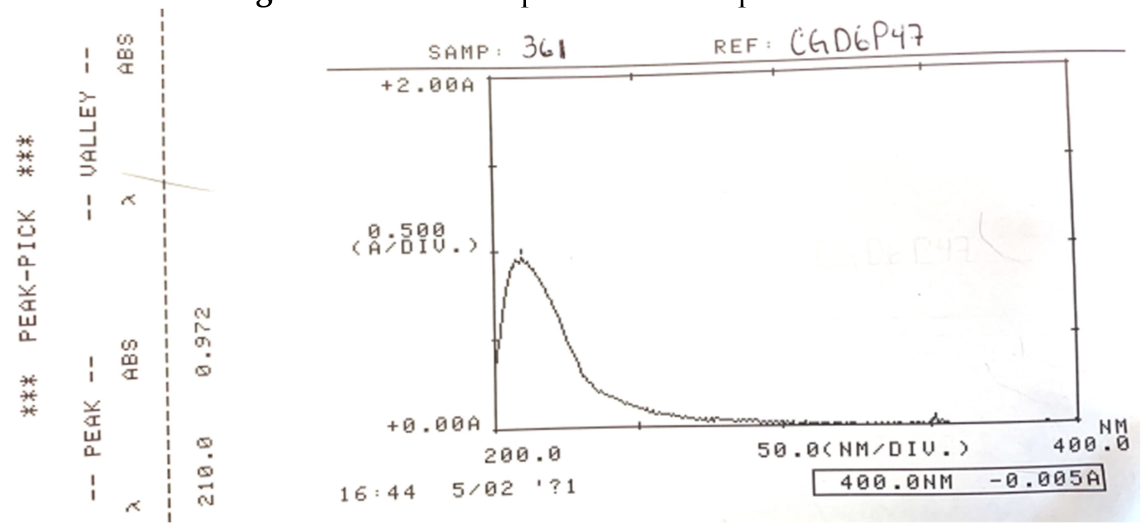

Figure S11. The IR spectrum of compound **2**.

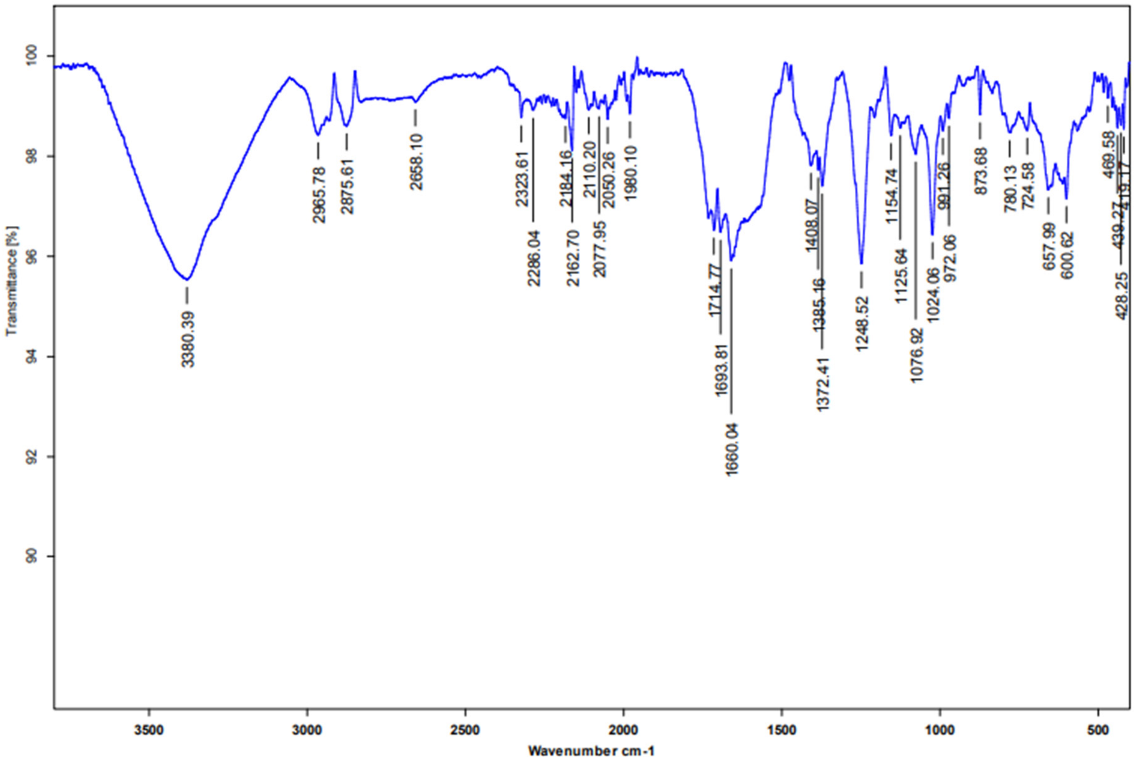

Figure S12. The ^{1}H NMR spectrum of **3** in CDCL3 (500 MHz).

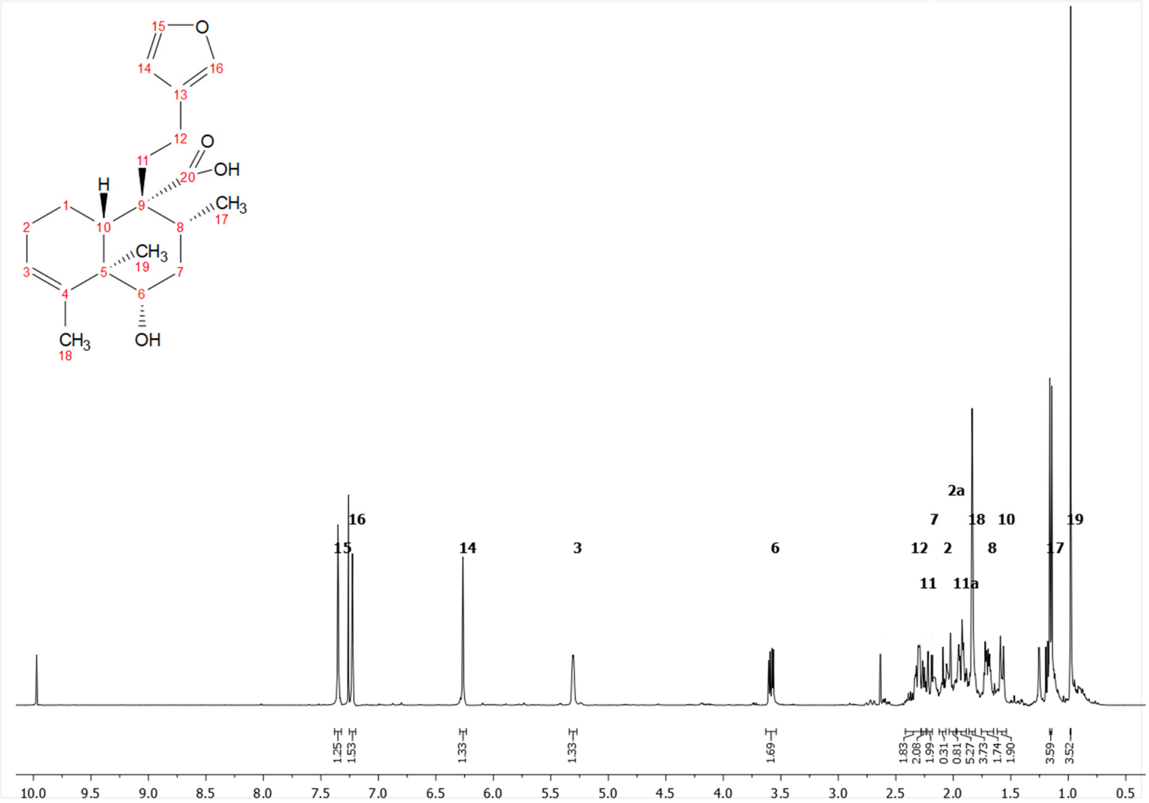

Figure S13. The ^{13}C NMR spectrum of **3** in CDCL3 (125 MHz).

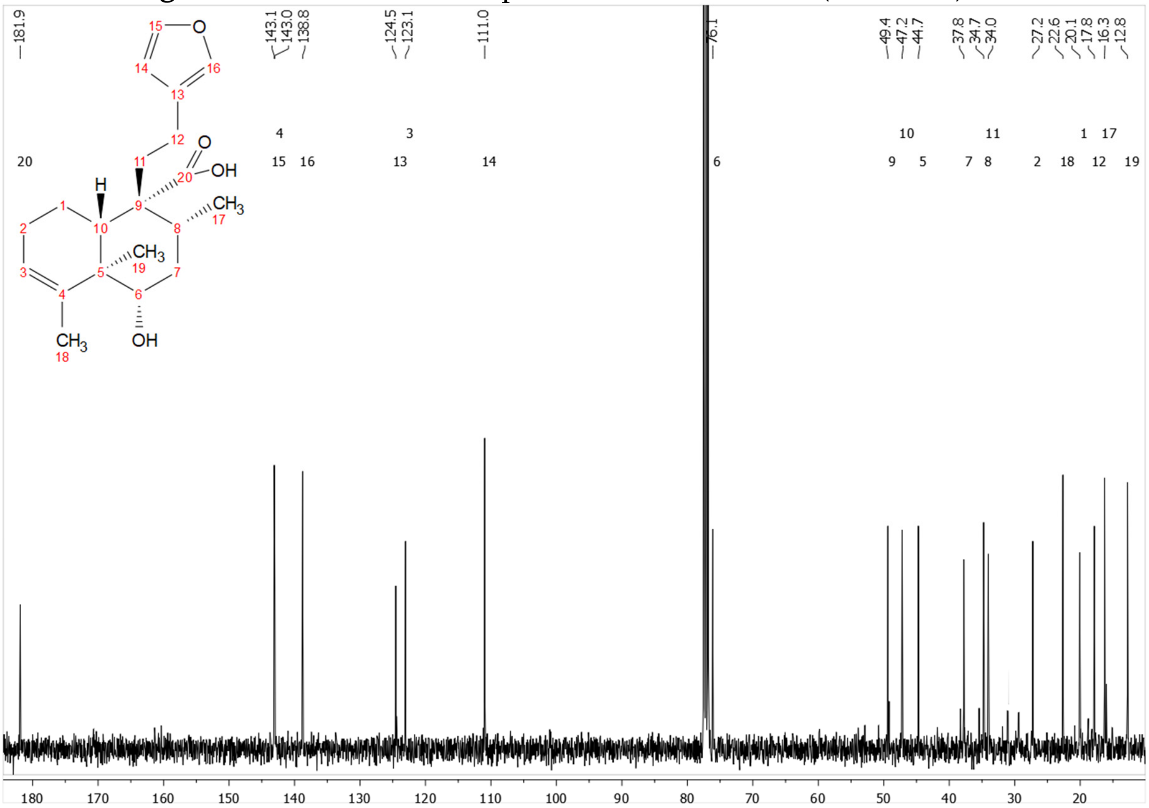

Figure S14. The DEPT spectrum of **3** in CDCL3 (125 MHz).

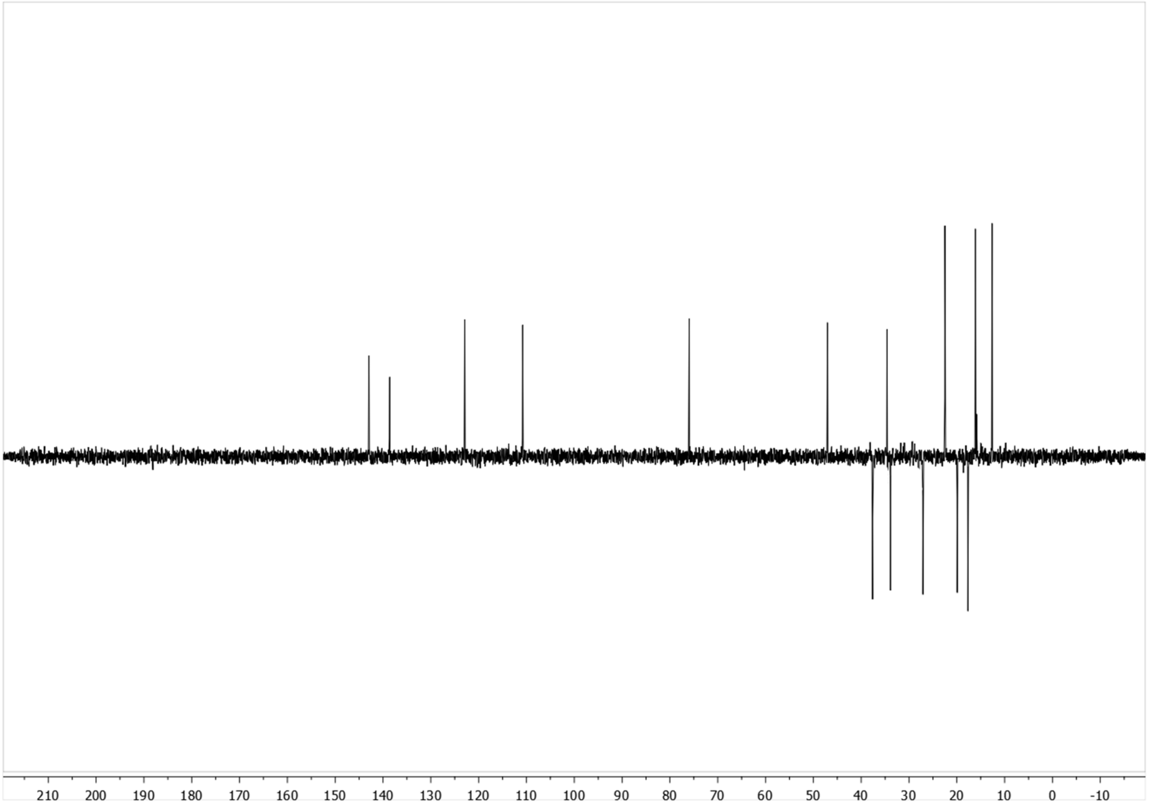

Figure S15. The HSQC spectrum of **3** in CDCL3.

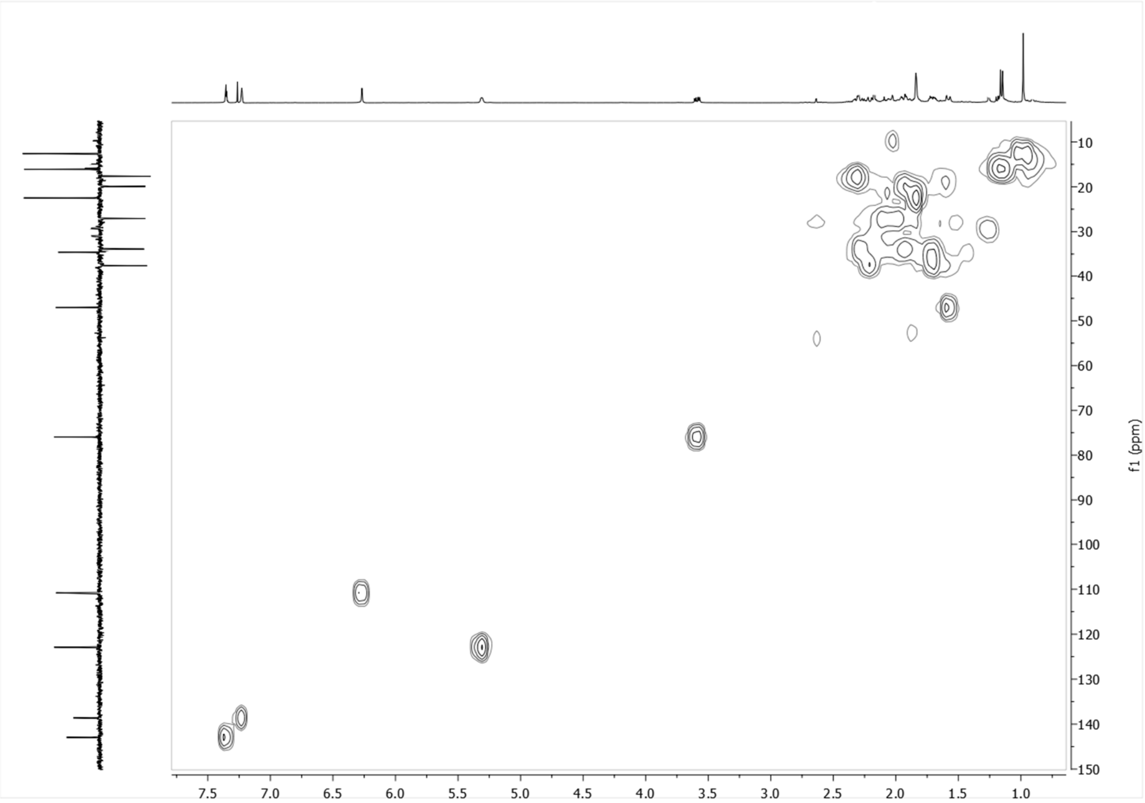

Figure S16. The ^1H-^1H COSY spectrum of **3** in CDCL$_3$.

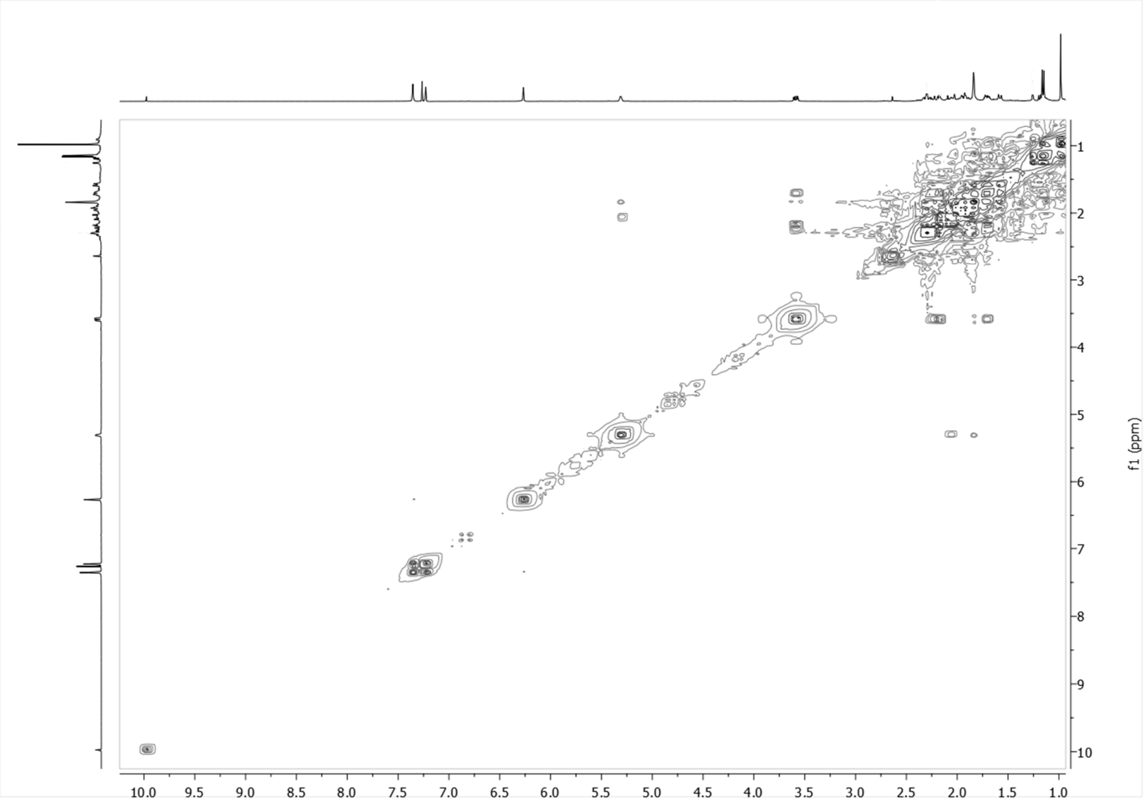

Figure S17. The HMBC spectrum of **3** in CDCL$_3$.

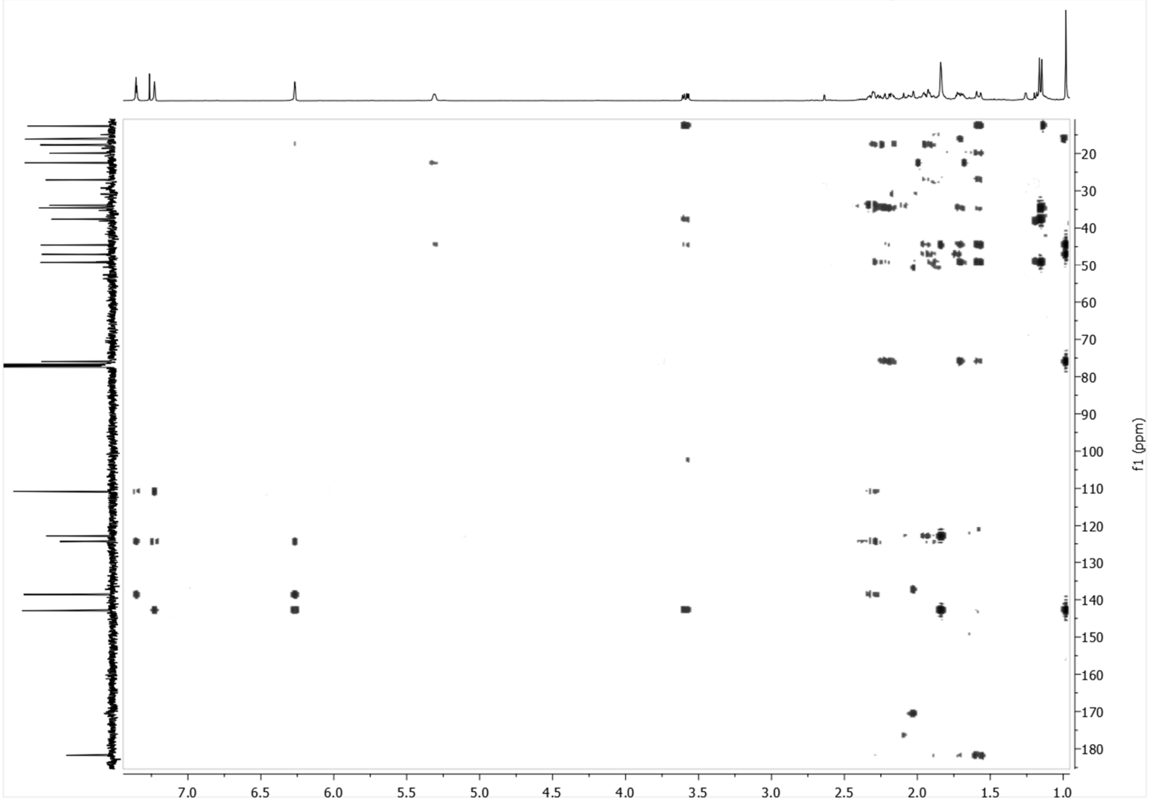

Figure S18. The NOESY spectrum of **3** in CDCL3.

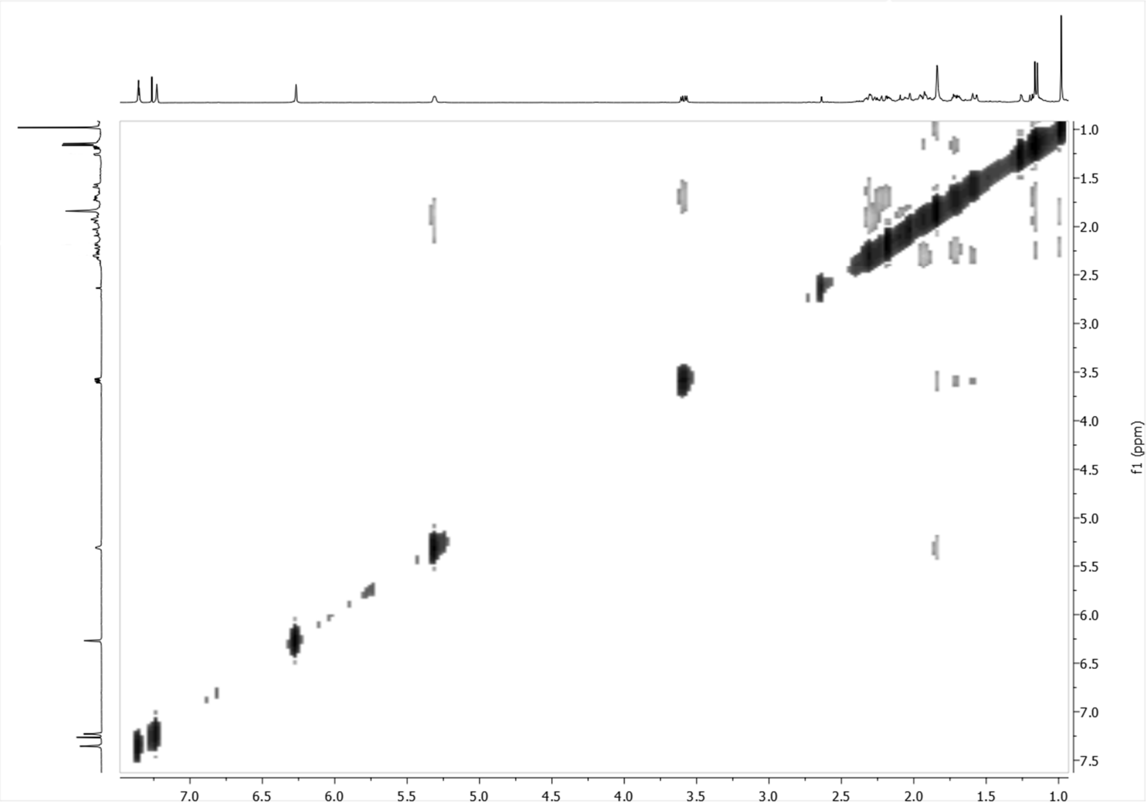

Figure S19. The TOCSY spectrum of **3** in CDCL3.

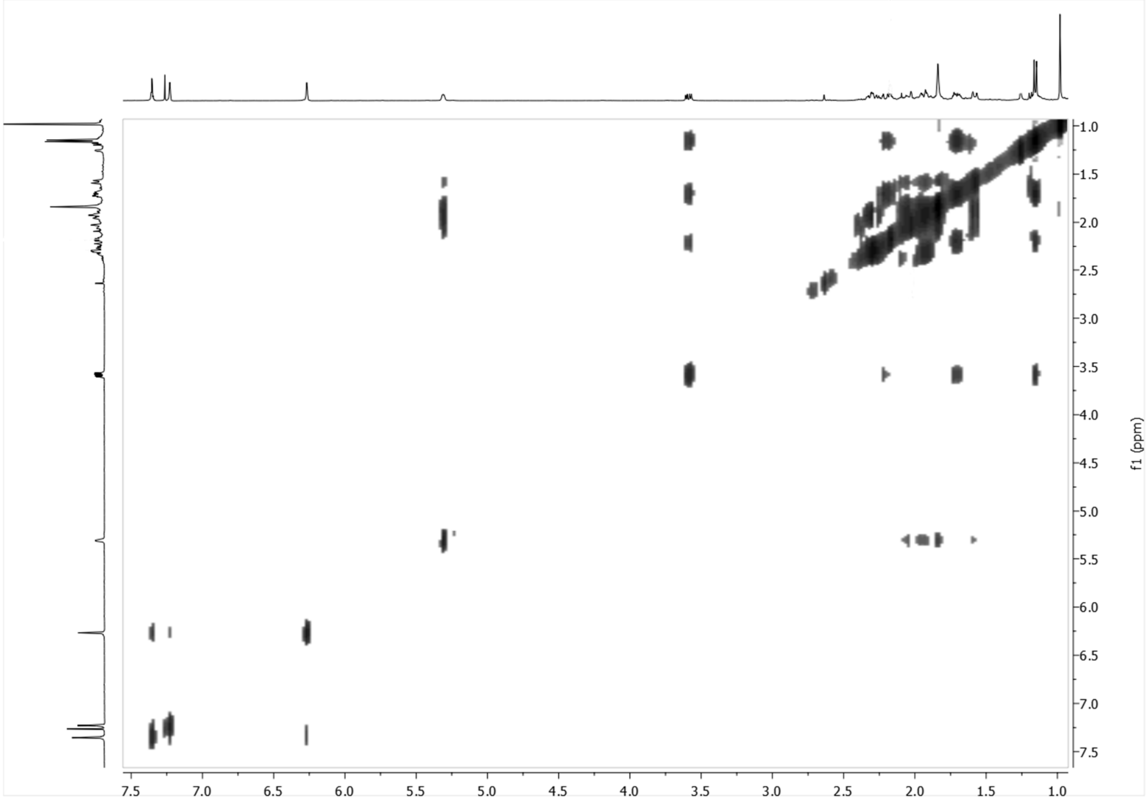

Figure S20. The HRESIMS spectrum of compound **3**.

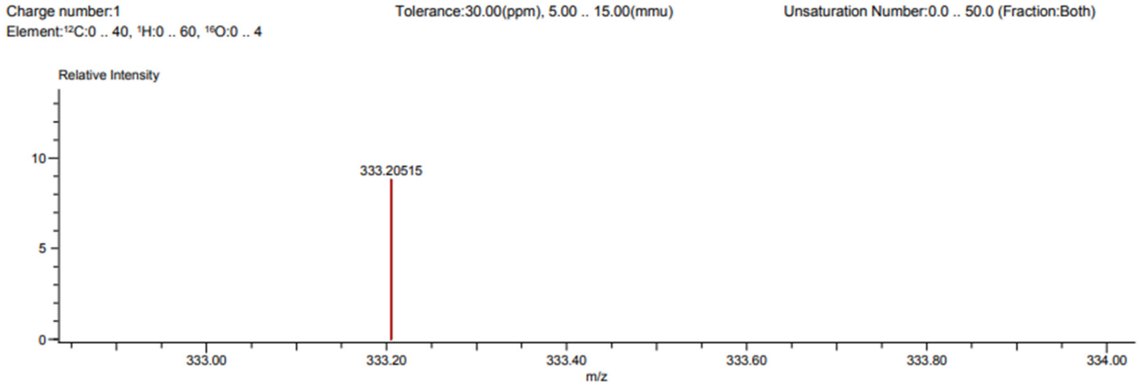

Mass	Intensity	Calc. Mass	Mass Difference (mmu)	Mass Difference (ppm)	Possible Formula	Unsaturation Number
333.20515	1747.37	333.20658	-1.43	-4.29	$^{12}C_{20}{}^{1}H_{29}{}^{16}O_{4}$	6.5

Figure S21. The UV spectrum of compound **3**.

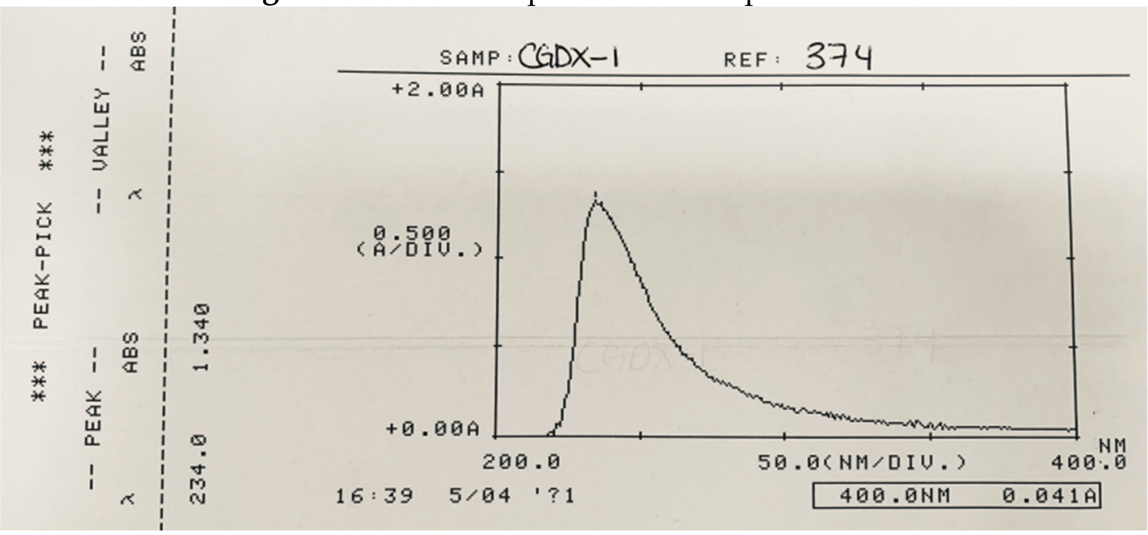

Figure S22. The IR spectrum of compound **3**.

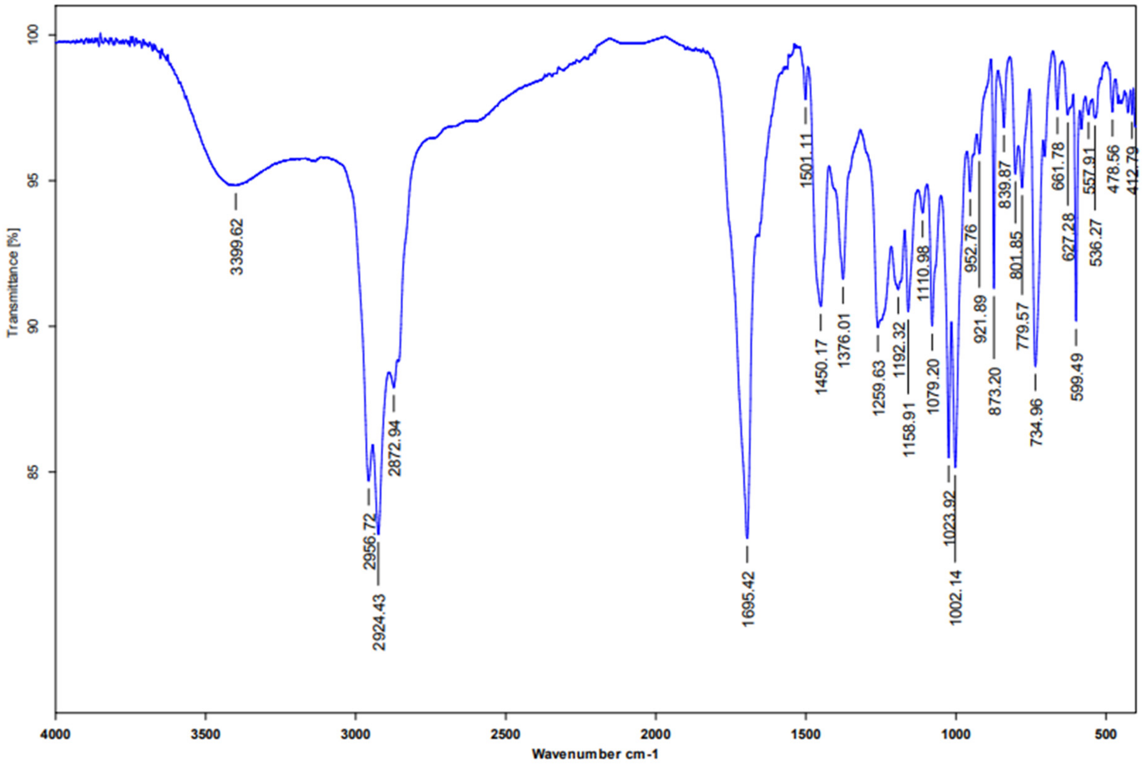

Figure S23. Calibration curve of rutin (**6**).

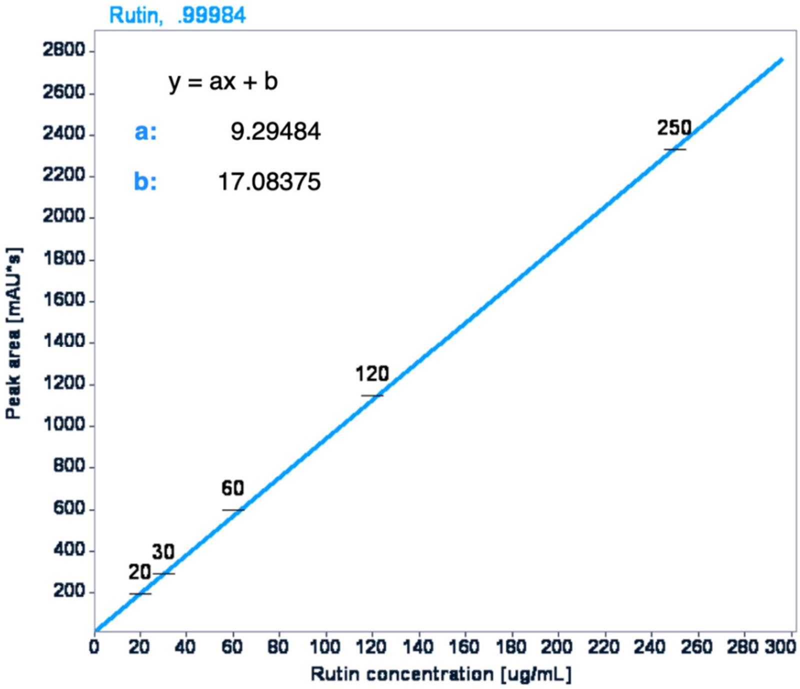

Figure S24. HPLC-MS spectrum obtained from the affinity screening analysis of EWE soluble extract with *a*-glucosidase, positive mode.

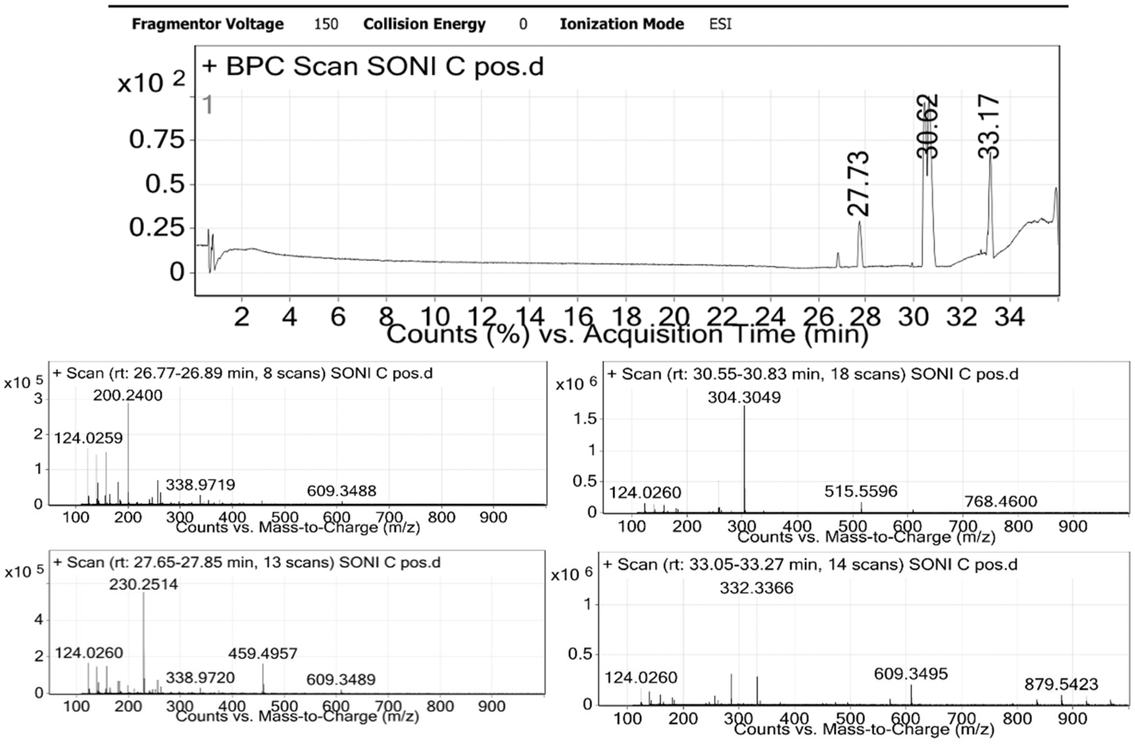

Figure S25. HPLC-MS spectrum obtained from the affinity screening analysis of EWE soluble extract with *a*-glucosidase, negative mode.

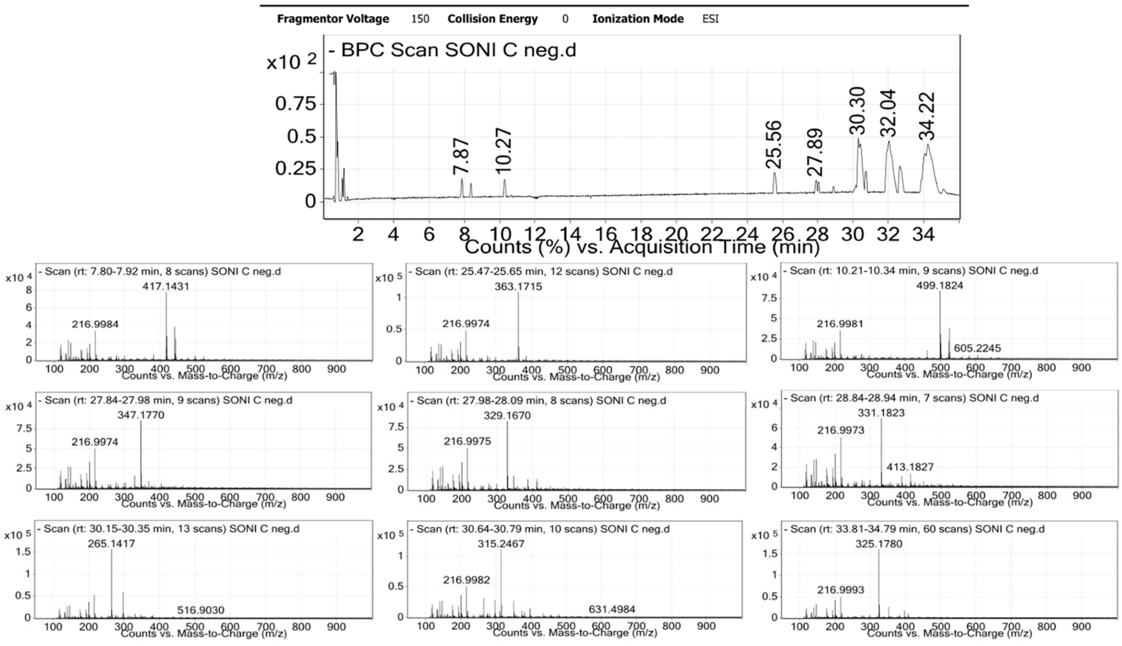

Table S1. Standard calibration curve of rutin (**6**).

Concentration (μg/mL)	Area (mAU)	Mean	%RSD[a]
	196.90		
20.00	193.10	196.53	1.66
	199.60		
	295.00		
40.00	295.40	294.37	0.50
	292.70		
	598.20		
80.00	591.20	597.07	0.90
	601.80		
	1131.70		
120.00	1149.70	1146.77	1.21
	1158.90		
	2311.10		
250.00	2341.80	2329.27	0.69
	2334.90		

[a] Relative Standard Deviation Percentage
